# Bowel cancer care in individuals with an intellectual disability: a population-based cohort study of symptoms, diagnostic pathways, treatment and survival

**DOI:** 10.1186/s12916-026-04906-9

**Published:** 2026-05-20

**Authors:** Oliver John Kennedy, Umesh Chauhan, Louise Gorman, Paul Lorigan, Samuel W. D. Merriel, Antonia Perumal, Tjeerd Van Staa, Alison Wright, Darren Mark Ashcroft

**Affiliations:** 1 Division of Cancer Sciences, University of Manchester, Manchester, UK; 2 The Christie NHS Foundation Trust, Manchester, UK; 3 School of Medicine, University of Lancashire, Lancashire, UK; 4 NIHR Greater Manchester Patient Safety Research Collaboration, University of Manchester, Manchester, UK; 5 Centre for Primary Care and Health Services Research, University of Manchester, Manchester, UK; 6 East Lancashire Hospitals NHS Trust, Royal Blackburn Hospital, Haslingden Road, Blackburn, Lancashire, BB2 3HH UK; 7 Centre for Pharmacoepidemiology and Drug Safety, Division of Pharmacy and Optometry, School of Health Sciences, Faculty of Biology, Medicine and Health, University of Manchester, Manchester, UK; 8 Manchester Academic Health Science Centre, Manchester, UK

**Keywords:** Learning disability, Intellectual disability, Bowel cancer, Bowel cancer screening

## Abstract

**Background:**

People with an intellectual disability (ID) are at increased risk of bowel cancer. However, evidence on their presenting symptoms, diagnostic pathways, treatments and survival remains limited.

**Methods:**

A matched cohort study was conducted using linked primary care (Clinical Practice Research Datalink), hospital, cancer, and mortality records. Outcomes included symptoms associated with bowel cancer, faecal immunochemical or faecal occult blood (FIT/FOB) testing, urgent suspected cancer (USC) referral, endoscopy, surgery, systemic anticancer therapy (SACT), and bowel cancer-specific mortality. Adjusted incidence rate ratios (aIRRs), risk ratios (aRRs), and hazard ratios (aHRs) were estimated using Poisson, modified Poisson and Cox regression.

**Results:**

A total of 111,034 individuals with an ID were matched to 1,964,420 comparators. ID was associated with increased risk of bowel cancer (aHR 1.30, 1.18–1.44), particularly before age 50 years (aRR 2.19, 1.68–2.85). People with an ID presented more frequently with symptoms associated with bowel cancer (aIRR 2.59, 2.53–2.65) but, following such symptoms, were less likely to undergo FIT/FOB testing (aRR 0.74, 0.67–0.83), USC referral (aRR 0.57, 0.52–0.62), endoscopy (aRR 0.45, 0.42–0.49), or receive a diagnosis within 56 days (aRR 0.52, 0.41–0.67). They were also less likely to be diagnosed via screening (aRR 0.27, 0.14–0.50) or USC referral (aRR 0.62, 0.50–0.76), and more likely to be diagnosed via emergency presentation (aRR 1.76, 1.52–2.02), on the date of death (aRR 5.08, 2.92–8.84), or with stage IV disease (aRR 1.25, 1.01–1.56). ID was associated with similar proportions receiving curative surgery for stage I–III disease (aRR 0.98, 0.79–1.19), but markedly lower proportions receiving SACT for stage IV (aRR 0.15, 0.05–0.46), and higher bowel cancer-specific mortality across all stages (aHR 2.00, 1.71–2.33).

**Conclusions:**

People with an ID experience worse outcomes across nearly all stages of the bowel cancer care pathway, including referral, investigation, treatment and survival. Earlier screening may be justified given the elevated risk in those under age 50 years.

## Background

An intellectual disability (ID) is a lifelong neurodevelopmental condition characterised by significant limitations in cognitive functioning and adaptive behaviours, such as social and practical skills, with onset during childhood [1]. Globally, approximately 1%–3% of the population, equating to around 200 million individuals, live with an ID, encountering unique challenges that impact daily life [2, 3]. This group frequently faces systemic barriers within healthcare services, including communication difficulties, inadequate provision of reasonable adjustments, and diagnostic overshadowing, where health issues are mistakenly attributed to the ID [3–5]. These challenges contribute to health inequalities, with adults with an ID experiencing a life expectancy reduction of 19–23 years compared to the general population, and nearly half of deaths amongst people with an ID deemed preventable through better care and timely interventions [6]. To tackle these disparities, initiatives such as the UK’s National Health Service (NHS) Long Term Plan (2019) and guidance from the National Institute for Health and Care Excellence (NICE) have prioritised improving equitable access to healthcare and enhancing health outcomes for individuals with an ID [7, 8].

ID is increasingly recognised as a hidden driver of cancer mortality [9–11]. While the relationship between ID and cancer varies by cancer type, studies consistently report a higher risk of bowel cancer and related deaths [12–14]. In addition, participation in the UK’s national bowel cancer screening programme is significantly lower among adults with an ID, with only 50.3% of eligible individuals taking part compared to 66.8% in the general population [15]. Reduced screening uptake may contribute to late-stage diagnoses and emergency presentations, though data on these outcomes are limited [16]. Further uncertainties exist, particularly regarding the risk of bowel cancer in younger individuals with an intellectual disability below 50 years of age, which is the age bowel cancer screening programmes begin in several countries. It also remains unclear how frequently symptoms associated with bowel cancer occur in this group, whether such symptoms are appropriately investigated in primary and secondary care settings, and how treatment decisions and survival outcomes compare following diagnosis.

The aim of this study was to examine the risk of bowel cancer in individuals with an ID, including those under the age of 50, and to assess the effect of an ID on each stage of the bowel cancer care pathway. This included the frequency of symptoms and patterns of investigation, such as faecal occult blood (FOB) or faecal immunochemical (FIT) testing, urgent suspected cancer referrals, endoscopy, surgery for stage I–III disease, systemic anticancer therapy for stage IV disease, and bowel cancer-specific survival, using a large population-based matched cohort.

## Methods

### Study population

The study cohort was from anonymised data held in the Clinical Practice Research Datalink (CPRD) Aurum database [17]. CPRD Aurum includes routinely recorded clinical information from general practices in England that use the EMIS Web system and is representative of the broader UK population in terms of age, gender and ethnicity [15]. General practice registration is necessary to access NHS healthcare and, as a result, almost all people in the UK are registered. On the date that data were extracted, CPRD Aurum contained information on approximately 50 million patients (including ~16 million currently contributing patients), representing around one-quarter of the UK population and one-fifth of general practices. In this study, primary care, ethnicity [18] and deprivation data were linked with mortality records from the Office for National Statistics (ONS), cancer registration data from the National Cancer Registration and Analysis Service (NCRAS), and secondary care data from Hospital Episode Statistics (HES).

### Inclusion and exclusion criteria

Participants eligible for inclusion were those with a documented diagnosis of an ID between 1 January 2000 and 31 December 2018. The date of the first recorded ID diagnosis within this time window served as the index date for matching purposes (further described below). A list of Read codes, SNOMED CT, and EMIS codes for intellectual disability were compiled based on previous research together with keyword searches of the CPRD data dictionary [19–28]. The list of codes comprised specific diagnostic codes for ID and codes related to conditions linked to ID (Additional File 1: Supplementary Table 1). This was because of known under-recording of ID in primary care, where only around 25% of individuals have a recorded diagnosis [29]. Codes relating to “learning difficulty” were considered equivalent to “learning disability” (another term for intellectual disability) in view of both terms being used interchangeably in UK legislation and NHS guidance [30]. The final list of codes was agreed on by two clinicians (OJK and UC), following previously applied methods [31]. Using this approach, the prevalence of ID in the CPRD population was 1.5%.

**Fig. 1:**
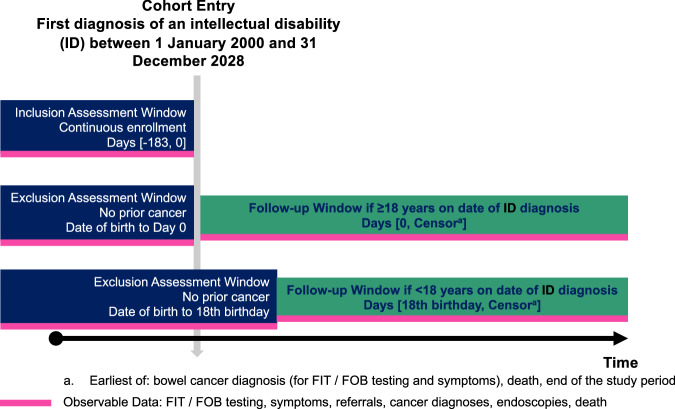
Cohort entry and availability of data for assessment of inclusion criteria, exclusion criteria and outcomes

Each person with an ID was matched with up to 20 comparators using incidence density sampling, matched by age (within a two-year range), gender, general practice and index date. Comparators had no recorded ID diagnosis before the index date and were given the same follow-up start date as their matched individual with an ID. Follow-up started on the later of the ID diagnosis date or the individual’s 18th birthday. This age was selected to balance the lower risk of bowel cancer in younger people with the need to capture early-onset disease. Early-onset bowel cancer is an emerging public health concern [32], and this study investigated whether it disproportionately affects people with an ID. Exclusion criteria included cancer diagnosis before follow-up, less than six months of continuous general practice registration before the index date, non-eligibility for linkage to the NCRAS, ONS, and HES datasets. Follow-up ended on 31 December 2018, the latest date with complete linked data. A standardised graphical overview of cohort entry and follow-up timelines is shown in Fig. 1 [33].

### Outcomes and statistical analysis

Pre-diagnostic outcomes of interest included symptoms potentially indicative of bowel cancer (Additional File 1: Supplementary Table 2) and faecal occult blood (FOB) or faecal immunochemical (FIT) testing. For these outcomes, each comparator’s follow-up was limited to the observation period of their matched case to allow for comparisons over equivalent timeframes. Outcomes were assessed following the first presentation with symptoms: within 28 days, these included completion of a FIT/FOB test (in the absence of a prior test in the preceding year) and urgent referral for suspected cancer; within 56 days, outcomes included endoscopy (e.g., colonoscopy or flexible sigmoidoscopy) and a confirmed diagnosis. Diagnoses within six-month, 1 and 3-year windows were additionally used to capture potential delays or missed diagnoses, including malignancy of undefined origin (MUO) and cancer of unknown primary (CUP), which may indicate late presentation or other barriers to investigation leading to undiagnosed bowel cancer. Among individuals diagnosed with bowel cancer, outcomes included stage at diagnosis (I, II, III, IV and unknown), route to diagnosis (screening programme, urgent suspected cancer referral and emergency presentation), receipt of curative surgery within six months for those with stages I-III disease and receipt of SACT within six months for those with stages IV disease. Associations between ID and diagnosis of bowel cancer at any age and before 50 years of age were also estimated.

Crude proportions for each outcome were summarised for individuals with and without an ID. Age, gender, ethnicity and deprivation-adjusted incidence rate ratios (aIRRs), risk ratios (aRRs) and hazard ratios (aHRs), along with 95% confidence intervals (CIs), were estimated using Poisson regression (with follow-up time as an offset in the model), modified Poisson regression with inverse probability of censoring weights, and Cox proportional hazards regression. Log-log (survival) versus log-time plots were used to check proportionality assumptions. Analyses of outcomes with follow-up starting at baseline (e.g., bowel cancer risk, rates of symptoms) used matched-set fixed effects or stratification with robust standard errors. Analyses with follow-up starting after a post-baseline event (e.g., bowel cancer-specific survival or outcomes following symptoms) used unmatched, event-anchored models to mitigate collider bias. Kaplan-Meier estimates were also used to estimate bowel cancer-specific survival. All statistical analyses were performed using R version 4.0.0. Missing data ( < 5% for all variables) were handled using multiple imputation by chained equations. The study was conducted in accordance with the RECORD (REporting of studies Conducted using Observational Routinely-collected health Data) guidelines [34].

## Results

The study population comprised 111,034 individuals with an ID and 1,964,420 matched comparators (Table 1). Among those with a recorded ID severity, 14,160 (12.8%), 14,119 (12.7%), and 9889 (8.9%), respectively, had mild, moderate and severe ID. Down syndrome was the cause of ID in 9390 patients (8.5%). The median ages at the start of follow-up were similar for those with and without an ID (34.0 vs. 35.8 years), as were the proportions of males (57.9% vs. 56.9%). A higher proportion of individuals with an ID were of White ethnicity compared to those without (86.3% vs. 82.6%), with lower proportions of unknown ethnicity (2.6% vs. 5.3%) and broadly similar proportions of Asian (5.6% vs. 6.6%) and Black ethnicities (4.0% vs. 3.8%). Individuals with an ID were more likely to live in areas of greater socioeconomic deprivation (27.2% vs. 22.8% in the most deprived quintile). The median follow-up was 4.8 years.

**Table 1:** Baseline characteristics of the included population

	No intellectual disability	Intellectual disability
**Total**	1,964,420	111,034
Gender		
Male	1,117,680 (56.9%)	64,268 (57.9%)
Female	846,740 (43.1%)	46,766 (42.1%)
**Age (years)**		
18–30	815,697 (41.5%)	49,219 (44.3%)
30–40	286,512 (14.6%)	15,689 (14.1%)
40–50	303,368 (15.4%)	16,339 (14.7%)
50–60	257,704 (13.1%)	13,732 (12.4%)
60–70	158,617 (8.1%)	8406 (7.6%)
>70	142,522 (7.3%)	7649 (6.9%)
**Ethnicity**		
White	1,622,952 (82.6%)	95,798 (86.3%)
Asian	129,220 (6.6%)	6169 (5.6%)
Black	75,192 (3.8%)	4479 (4%)
Mixed/Multiple	25,567 (1.3%)	1566 (1.4%)
Other	7630 (0.4%)	182 (0.2%)
Unknown	103,859 (5.3%)	2840 (2.6%)
**Deprivation (IMD quintile)**		
1 - Least deprived	347,257 (17.7%)	14,824 (13.4%)
2	370,388 (18.9%)	18,721 (16.9%)
3	380,329 (19.4%)	21,651 (19.5%)
4	415,110 (21.1%)	25,506 (23%)
5 - Most deprived	448,408 (22.8%)	30,169 (27.2%)
Unknown	2928 (0.1%)	163 (0.1%)

*IMD* Index of Multiple Deprivation

As shown in Table 2, ID was associated with a significantly higher incidence rate of symptoms (IRR 2.59, 95% CI 2.53–2.64) but a reduced incidence rate of FIT/FOB testing (IRR 0.92, 95% CI 0.91–0.93). Following their first symptom, individuals with an ID were markedly less likely to have a FIT or FOB test (RR 0.74, 95% CI 0.67–0.83, Table 3) or an urgent suspected cancer referral (RR 0.57, 95% CI 0.52–0.62) within 28 days. They were also less likely to have an endoscopy (RR 0.45, 95% CI 0.42–0.49) or receive a diagnosis within 56 days (RR 0.52, 95% CI 0.41–0.67). Differences in diagnosis decreased over time: at six months (RR 0.64, 95% CI 0.53–0.77), one year (RR 0.67, 95% CI 0.56–0.79), and three years (RR 0.78, 95% CI 0.68–0.90) after the first symptom. When MUO and CUP cases were included, the difference at three years was not statistically significant (RR 0.89, 95% CI 0.78–1.01).

**Table 2:** Associations of intellectual disability with bowel cancer, symptoms suggestive of bowel cancer and FIT / FOB testing

	No ID	ID	Estimate
**Bowel cancer risk**	Diagnoses / total	Diagnoses / total	HR (95% CI)
	6936 / 1,964,420	381 / 111,034	1.30 (1.18-1.44)
**Bowel cancer risk age** < **50 years**	Diagnoses <50 years / total <50 years	Diagnoses <50 years / total <50 years	RR (95% CI)
	489 / 1,405,445	58 / 81,240	2.19 (1.68-2.85)
**Symptoms suggestive of bowel cancer**	Patients with symptoms^a^ / total patients	Patients with symptoms / total patients	IRR (95% CI)
	307,560 / 1,964,420	35,247 / 111,034	2.59 (2.53-2.65)
**FIT / FOB test**	Patients tested / total patients	Patients tested / total patients	IRR (95% CI)
	208,723 / 1,964,420	12,793 / 111,034	0.92 (0.91-0.93)

*ID* intellectual disability, *FIT* faecal immunochemical test, *FOB* faecal occult blood test, *HR* hazard ratio, *RR* risk ratio, *IRR* incidence rate ratio, *CI* confidence interval

^a^Follow-up truncated to that of corresponding case

**Table 3:** Associations between intellectual disability and outcomes following a first symptom suggestive of bowel cancer

	No ID (N)	ID (N)	RR (95% CI)
Total patients with ≥1 symptom	405,891	35,427	
FIT / FOB test within 28 days of first symptom	5070	333	0.74 (0.67–0.83)
Referral within 28 days of first symptom	9513	479	0.57 (0.52–0.63)
Endoscopy within 56 days of first symptom	17,324	731	0.45 (0.42–0.49)
Diagnosis within 56 days of first symptom	1448	65	0.52 (0.41–0.67)
Diagnosis within 6 months of first symptom	2154	117	0.64 (0.53–0.77)
Diagnosis within 1 year of first symptom	2462	137	0.67 (0.56–0.79)
Diagnosis within 3 years of first symptom	3035	192	0.78 (0.68–0.90)
Diagnosis within 3 years (inc. MUO / CUP)	3411	244	0.89 (0.78–1.01)

*ID* intellectual disability, *FIT* faecal immunochemical test, *FOB* faecal occult blood test, *RR* risk ratio, *CI* confidence interval, *MUO* malignancy of undefined origin, *CUP* cancer of unknown primary

During follow-up, 381 individuals with an ID and 6936 without were diagnosed with bowel cancer, including 58 and 489 cases, respectively, under the age of 50. ID was strongly associated with a higher overall risk of bowel cancer (HR 1.30, 95% CI 1.18–1.44, Table 2) and a twofold increase in risk before age 50 (RR 2.19, 95% CI 1.68–2.85). Diagnoses through screening programmes were less common among individuals with an ID (*n* = 10 [2.9%] vs. *n* = 580 [10.4%]; RR 0.27, 95% CI 0.14–0.50), as were diagnoses via urgent suspected cancer referrals (*n* = 72 [20.6%] vs. *n* = 1896 [34.0%]; RR 0.62, 95% CI 0.50–0.76) (Table 4). In contrast, emergency presentations were more common in the ID group (*n* = 136 [39.0%] vs. *n* = 1240 [22.3%]; RR 1.76, 95% CI 1.52–2.02), as was having the diagnosis first recorded on the date of death (*n* = 16 [4.2%] vs. *n* = 60 [1.0%]; RR 5.08, 95% CI 2.92–8.84) and stage IV disease at diagnosis (*n* = 89 [23.4%] vs. *n* = 1178 [19.4%]; RR 1.25, 95% CI 1.01–1.56).

**Table 4:** Associations between intellectual disability and outcomes among those diagnosed with bowel cancer

	No ID	ID	RR (95% CI)
**Route to diagnosis**	**N / total diagnoses**^a^	**N / total diagnoses**^a^	
Screening	10 / 349 (2.9%)	580 / 5571 (10.4%)	0.27 (0.14-0.50)
Urgent suspected cancer referral	72 / 349 (20.6%)	1896 / 5571 (34.0%)	0.62 (0.50-0.76)
Emergency presentation	136 / 349 (39.0%)	1240 / 5571 (22.3%)	1.76 (1.52-2.02)
**Diagnosis recorded on date of death**	**N / total diagnoses**	**N / total diagnoses**	
	60 / 6085 (1.0%)	16 / 381 (4.2%)	5.08 (2.92-8.84)
**Curative surgery within 6 months**	**N / total with non-metastatic disease**^b^	**N / total with non-metastatic disease**^b^	
	1813 / 2218 (81.7%)	95 / 118 (80.5%)	0.98 (0.79-1.19)
**SACT within 6 months for metastatic disease**	**N / total with metastatic disease**^b,c^	**N / total with metastatic disease**^b,c^	
	241 / 476 (50.6%)	<5 / 34	0.15 (0.05-0.46)
**Stage at diagnosis**	**N**	**N**	
Stage I	736 (12.1%)	36 (9.4%)	
Stage II	979 (16.1%)	53 (13.9%)	
Stage III	1179 (19.4%)	69 (18.1%)	
Stage IV	1178 (19.4%)	89 (23.4%)	1.25 (1.01-1.56)^d^
Unknown	2013 (33.1%)	134 (35.2%)	1.08 (0.91-1.29)^e^

*ID* intellectual disability, *FIT* faecal immunochemical test, *FOB* faecal occult blood test, *RR* risk ratio, *CI* confidence interval, *SACT* systemic anticancer therapy

^a^With recorded route to diagnosis

^b^With required follow-up

^c^From 2014 due to availability of SACT data

^d^Stage 4 vs. stages 1–3

^e^Known stage vs. unknown stage

**Fig. 2:**
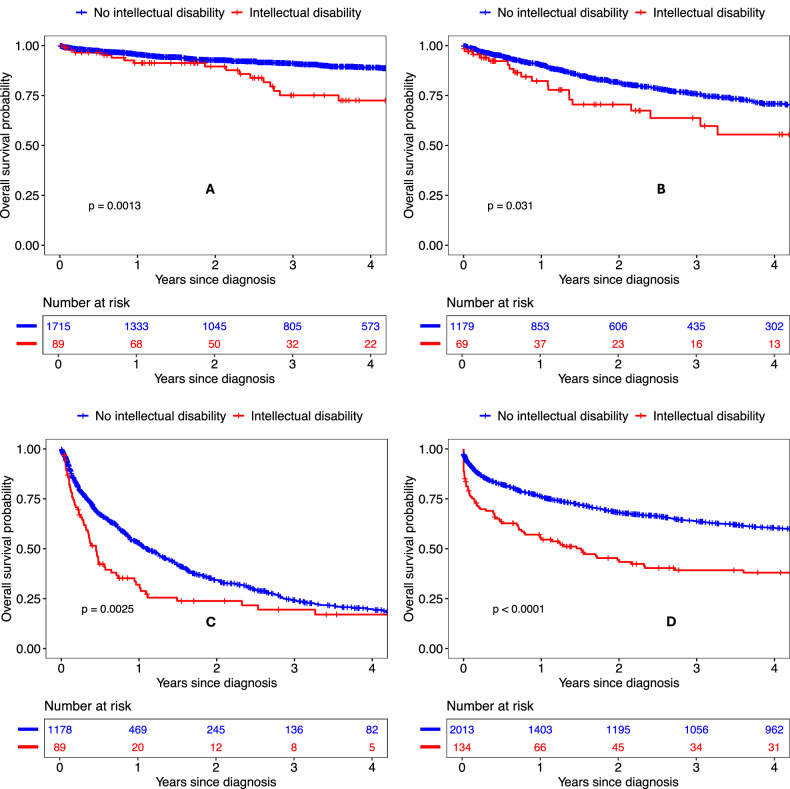
Bowel cancer specific survival among individuals with and without an intellectual disability with stage 1-2 (**A**), 3 (**B**), 4 (**C**) and unknown (**D**) stage disease

A total of 95 (80.5%) individuals with an ID underwent curative surgery for stage I-III disease within six months (Table 4), compared with 1813 (81.7%) without an ID (RR 0.98, 95% CI 0.79–1.19). In comparison, fewer than five individuals with an ID and 241 (50.6%) without an ID received SACT within six months of diagnosis for stage IV disease (RR 0.15, 95% CI 0.05–0.46). Among all those diagnosed, ID was associated with shorter bowel cancer-specific survival (Table 5; HR 2.00, 95% CI 1.71–2.33). This pattern was consistent across all stages (Fig. 2), with particularly pronounced differences in three-year survival for stage I-II disease (75.1% [95% CI 61.4–84.5] for people with an ID vs. 91.1% [95% CI 89.5–92.5] for those without) and one-year survival for stage IV disease (32.0%, 95% CI 21.8–42.7 vs. 52.9%, 95% CI 49.8–56.0).

**Table 5:** Association between intellectual disability and bowel cancer specific survival

	No ID (deaths / total)	ID (deaths / total)	1-year (no ID)	1-year (ID)	3-year (no ID)	3-year (ID)	HR (95% CI)
All	2015 / 6085	176 / 381	80.1% (79.1–81.1)	63.1% (57.8–68.0)	66.6% (65.3–67.9)	47.6% (41.7–53.3)	2.00 (1.71-2.33)
Stages 1–2	162 / 1715	17 / 89	95.5% (94.4–96.5)	91.3% (82.5–95.8)	91.1% (89.5–92.5)	75.1% (61.4–84.5)	2.34 (1.40-3.90)
Stage 3	270 / 1179	19 / 69	90.3% (88.4–91.9)	82.3% (69.3–90.1)	75.6% (72.5–78.4)	63.7% (47.1–76.4)	2.13 (1.33-3.41)
Stage 4	748 / 1178	61 / 89	52.9% (49.8–56.0)	32.0% (21.8–42.7)	24.2% (21.2–27.3)	19.5% (10.8–30.1)	1.63 (1.25-2.13)
Unknown	835 / 2013	79 / 134	76.1% (74.1–77.9)	54.6% (45.6–62.7)	63.7% (61.5–65.9)	39.2% (30.3–48.0)	2.27 (1.79-2.86)

*ID* intellectual disability, *HR* hazard ratio, *CI* confidence interval

## Discussion

In this large population-based matched cohort study of over two million individuals, the impact of an ID on the bowel cancer care pathway was assessed. Individuals with an ID were more likely to develop bowel cancer, particularly those under the age of 50. They were also more likely to present to primary care with symptoms associated with bowel cancer but, following such symptoms, were less likely to receive timely investigations, including FIT/FOB testing, referral, and endoscopy. They were less likely to receive a diagnosis of bowel cancer within 56 days, though the total proportions diagnosed with either bowel cancer or MUO/CUP were similar after three years. Bowel cancer was less often diagnosed through screening or urgent suspected cancer referral, and more often diagnosed following an emergency presentation, with stage IV disease, or first recorded on the date of death. ID was associated with reduced likelihood of SACT for stage IV disease, and bowel cancer-specific survival was significantly poorer across all stages.

Considering growing concern about the rising incidence of bowel cancer in younger people, the finding of a twofold higher risk of bowel cancer in individuals with an ID under the age of 50 warrants further investigation. Early-onset bowel cancer is poorly understood, but proposed risk factors include obesity, processed diet, and physical inactivity [35], which may be more common in individuals with an ID [36, 37]. As such, individuals with an ID may be disproportionately affected by this trend of increasing early-onset bowel cancer as well as being an under-recognised contributor to it. The elevated risk in those under 50 also indicates that current bowel cancer screening programmes, which in many countries do not begin until age 50, may not be providing equitable benefit for people with an ID, and earlier screening in this population may be merited.

This study identifies specific stages within the bowel cancer diagnostic pathway where disparities may contribute to poorer outcomes for individuals with an ID. Lower rates of FIT/FOB testing, urgent referrals, and endoscopic procedures may result in underdiagnosis and explain the greater likelihood of bowel cancer being identified during emergency presentations, at stage IV disease, or only on the date of death. The reasons for these missed opportunities for investigation remain unclear; however, clinicians may face challenges due to the higher prevalence of bowel-related symptoms in individuals with ID, making it harder to distinguish potentially concerning signs. Additional concerns may include issues around consent or perceived fitness for endoscopy or treatment if cancer is diagnosed. Nonetheless, the findings suggest that improving the investigation of symptoms in this population could facilitate earlier detection and lead to better outcomes.

It should also be noted that while differences in the proportion diagnosed after symptom presentation may indicate under-investigation, they may also reflect reduced specificity of guideline-listed bowel cancer symptoms in individuals with an intellectual disability. In the current study, the proportions diagnosed with bowel cancer in the two groups became more similar over time and were comparable by three years when MUO and CUP cases were included. These may represent bowel cancers that could not be fully investigated. This suggests that although reduced symptom specificity may play a role, under-investigation is likely a major contributing factor.

Among individuals diagnosed with stages I-III disease, receipt of curative surgery within six months was similar by ID status. However, bowel cancer-specific survival was substantially worse in individuals with an ID. Given similar curative surgery rates, this survival gap may be driven by delays in diagnosis and a higher frequency of emergency presentations, which can limit the time available for complete diagnostic workup (e.g. full staging) and coordinated treatment planning. Surgical outcomes may also differ in patients with and without an ID, for example if there were differences in procedure selection (e.g., total mesorectal excision vs transanal approaches due to concerns about morbidity), peri-operative SACT use, complication rates, or postoperative care. For patients with stage IV disease, those with an ID were more than 80% less likely to receive SACT and, accordingly, had markedly shorter survival. The reasons for this are unclear but may include concerns about fitness, capacity, or the ability to self-report adverse events such as fever due to neutropenic sepsis, which carries a high risk of mortality if not promptly treated.

Several earlier studies have investigated bowel cancer outcomes in individuals with an ID, although information on presentation, investigations, referrals, and treatment remains very limited. Most previous studies, similar to the present study, have reported worse bowel cancer-related outcomes in this population. In a cross-sectional study in the Netherlands involving around 100,000 individuals with an ID, Banda et al. [38] reported lower participation in bowel cancer screening (51.7% vs. 72.7%; odds ratio [OR] 0.40, 95% CI 0.41–0.42). In the UK, Heslop et al. [16] analysed the death records of 1096 adults and found that 36% of cancer deaths in people with ID were due to digestive system cancers, with bowel cancer accounting for roughly half of these. A higher burden of bowel cancer relative to other cancer types in this population, compared with the general population, may be expected given higher rates of obesity but lower rates of smoking and alcohol use [36, 37]. A Scottish study found similar incidence rates of bowel cancer in people with and without ID, but higher mortality in the ID group (standardised mortality ratio 1.54) [39]. A Canadian study by Mahar et al. [13] found that individuals with intellectual and developmental disabilities diagnosed with bowel cancer were 1.44 times more likely to have stage IV disease at diagnosis compared to those without. Another study in Canada [12] found that ID was associated with a 2.42-fold higher risk of death in patients with bowel cancer. A review by Willis et al. [40] reported that diagnosis was frequently delayed due to communication difficulties and limited awareness among health and social care staff. In the United States, an analysis of over 722,000 patients undergoing bowel cancer surgery showed that those with an ID had significantly worse outcomes, including a twofold higher risk of in-hospital mortality, more complications, and substantially greater odds of non-home discharge [41].

Several limitations of this study should be acknowledged. The observational design precludes conclusions about causality. Although matching and adjustment for age and gender reduced confounding, residual confounding cannot be ruled out. The representativeness of our ID cohort may have been affected because some individuals with ID may not have a recorded diagnosis in their primary care record. Using primary care records, our estimated ID prevalence was 1.5%, which was below the estimated 2.13% in England [42]. If missing patients were predominantly people with mild ID, and if mild ID had a smaller effect on the outcomes than more severe ID, then their exclusion may have exaggerated the observed association between ID and the outcomes. Conversely, if some of these individuals were misclassified into the non-ID group, this may have attenuated the observed associations. While the large overall sample size enabled robust comparisons, subgroup analyses were limited due to under recording of some covariates or smaller sample sizes (e.g., for ethnicity, deprivation, severity of ID, underlying ID aetiology, and residential status). The dataset lacked clinical context necessary to assess the appropriateness of individual decisions along the care pathway. For instance, although individuals with an ID were less likely to receive FIT/FOB testing after presenting with symptoms suggestive of bowel cancer, we could not determine whether this reflected appropriate clinical judgment based on the nature, severity, or persistence of symptoms. We also did not differentiate between FIT/FOB tests performed as part of the national screening programme and those performed to investigate symptoms. Our findings of lower FIT/FOB tests rates in ID following symptoms may therefore have been influenced by the known lower participation in screening programmes. Similarly, we were unable to assess whether lower rates of investigation or diagnosis were due to clinical ineligibility (e.g., fitness for endoscopy) or other system- or patient-level barriers. Our analysis including MUO/CUP cases, which aimed to capture potentially missed bowel cancers, was limited by the possibility that it also encompassed malignancies not of bowel origin. We were unable to investigate the role of perioperative SACT or chemoradiotherapy in stages I-III disease.

Future research should explore how clinical decision-making, patient characteristics, and organisational barriers may contribute to observed differences in diagnosis and treatment outcomes. Further work is also needed to examine the potential benefits of lowering the bowel cancer screening age for people with ID, as recommended by the LEDER report [43]. This is important given our findings of an approximately two-fold higher risk of bowel cancer before age 50 (i.e., the age screening typically begins) in this group. Such work should include cost-effectiveness modelling to determine under which conditions a younger screening age might be justified, taking into account screening costs per person with ID, expected uptake rates, and the sensitivity and specificity of testing in younger patients. Feasibility considerations would also need to be addressed, including the reliable identification of ID in health records and potential heterogeneity in risk and outcomes by severity of ID and the presence of concomitant physical disability. The annual Learning Disability Health Check may offer a practical opportunity to assess screening status and deliver interventions aimed at improving bowel cancer diagnosis among people with ID. Further research is needed to evaluate whether such an approach would be feasible and effective in practice.

## Conclusions

This large, population-based cohort study reveals marked disparities across the bowel cancer diagnostic and care pathway for individuals with an ID. Despite being more likely to report symptoms suggestive of bowel cancer, they were less likely to receive timely investigations, such as FIT/FOB testing, referrals, or endoscopy, and more likely to be diagnosed during emergency presentations or at the time of death. Key diagnostic steps were often missed or delayed, potentially contributing to poorer outcomes. Focused efforts to investigate the causes of these gaps and to develop tailored strategies to improve early detection and care are urgently needed to reduce inequities for people with an ID.

## Supplementary information

Additional File 1: Supplementary Table 1. A list of codes to identify intellectual disabilities; Supplementary Table 2. A list of codes to identify symptoms testing of potentially indicative of bowel cancer

## Data Availability

Electronic health records are, by definition, considered sensitive data in the UK by the Data Protection Act and cannot be shared via public deposition because of information governance restriction in place to protect patient confidentiality. Access to Clinical Practice Research Datalink (CPRD) data is subject to protocol approval via CPRD’s research data governance process. For more information see https://cprd.com/data-access. Linked secondary care data from Hospital Episodes Statistics, mortality data from the Office for National Statistics, cancer data from the National Cancer Registration and Analysis Service and index of multiple deprivation data can also be requested from CPRD.

## References

[1] National Institute for Health and Care Excellence. Clinical Knowledge Summaries. Learning Disabilities. 2023. https://cks.nice.org.uk/topics/learning-disabilities/background-information/definition. Accessed 27 May 2025.

[2] Nair R, Chen M, Dutt AS, Hagopian L, Singh A, Du M. Significant regional inequalities in the prevalence of intellectual disability and trends from 1990 to 2019: a systematic analysis of GBD 2019. Epidemiol Psychiatr Sci. 2022;31:e91. 36539341 10.1017/S2045796022000701 PMC9805697

[3] Parkin, E House of commons library. research briefing. Learning disabilities: health policies. https://researchbriefings.files.parliament.uk/documents/SN07058/SN07058.pdf. Accessed 1 May 2025.

[4] Kennedy OJ, Chauhan U, Gorman L, Lorigan P, Merriel SWD, Staa TV, et al. Prostate cancer care for men with an intellectual disability: a population-based cohort study of symptoms, diagnosis, treatment, and survival. Eur Urol Oncol. 2026. 10.1016/j.euo.2026.01.004. Online ahead of print. 10.1016/j.euo.2026.01.004 41720694

[5] Kennedy OJ, Chauhan U, Gorman L, Lorigan P, Merriel S, Van Staa T, et al. Cancer diagnoses, referrals, and survival in people with a learning disability in the UK: a population-based, matched cohort study. Lancet Reg Health Eur. 2025;60:101519. 41497315 10.1016/j.lanepe.2025.101519 PMC12767837

[6] NHS England. Learning from lives and deaths - people with a learning disability and autistic people (LeDeR). Action from learning report 2022/23. https://leder.nhs.uk/images/resources/action-from-learning-report-22-23/20231019_LeDeR_action_from_learning_report_FINAL.pdf. Accessed 1 May 2025.

[7] NHS England. The NHS long term plan. 2019. www.longtermplan.nhs.uk/publication/nhs-long-term-plan. Accessed 21 May 2025.

[8] National Institute for Health and Care Excellence. NICE guideline: NG96. Care and support of people growing older with learning disabilities. 2018. https://www.nice.org.uk/guidance/ng96/chapter/recommendations. Accessed 1 May 2025.

[9] Glover G, Williams R, Heslop P, Oyinlola J, Grey J. Mortality in people with intellectual disabilities in England. J Intellect Disabil Res. 2017;61:62–74. 27489075 10.1111/jir.12314

[10] Banda A, Naaldenberg J, Timen A, van Eeghen A, Leusink G, Cuypers M. Cancer risks related to intellectual disabilities: a systematic review. Cancer Med. 2024;13:e7210. 38686623 10.1002/cam4.7210 PMC11058689

[11] The Lancet O. Intellectual and developmental disabilities-an under-recognised driver of cancer mortality. Lancet Oncol. 2024;25:411. 38547885 10.1016/S1470-2045(24)00146-3

[12] Hansford RL, Ouellette-Kuntz H, Griffiths R, Hallet J, Decker K, Dawe DE, et al. Breast (female), colorectal, and lung cancer survival in people with intellectual or developmental disabilities: a population-based retrospective cohort study. Can J Public Health. 2024;115:332–42. 38315327 10.17269/s41997-023-00844-8 PMC11027730

[13] Mahar AL, Biggs K, Hansford RL, Derksen S, Griffiths R, Enns JE, et al. Stage IV breast, colorectal, and lung cancer at diagnosis in adults living with intellectual or developmental disabilities: a population-based cross-sectional study. Cancer. 2024;130:740–9. 37902956 10.1002/cncr.35068

[14] Liu Q, Adami HO, Reichenberg A, Kolevzon A, Fang F, Sandin S. Cancer risk in individuals with intellectual disability in Sweden: a population-based cohort study. PLoS Med. 2021;18:e1003840. 34673770 10.1371/journal.pmed.1003840 PMC8568154

[15] NHS England. Health and care of people with learning disabilities, experimental statistics 2021 to 2022. https://digital.nhs.uk/data-and-information/publications/statistical/health-and-care-of-people-with-learning-disabilities/experimental-statistics-2021-to-2022. Accessed 12 July 2025.

[16] Heslop P, Cook A, Sullivan B, Calkin R, Pollard J, Byrne V. Cancer in deceased adults with intellectual disabilities: english population-based study using linked data from three sources. BMJ Open. 2022;12:e056974. 35332044 10.1136/bmjopen-2021-056974 PMC8948391

[17] Wolf A, Dedman D, Campbell J, Booth H, Lunn D, Chapman J, et al. Data resource profile: clinical practice research datalink (CPRD) aurum. Int J Epidemiol. 2019;48:1740. -g 30859197 10.1093/ije/dyz034 PMC6929522

[18] Medicines and Healthcare products Regulatory Agency. Clinical Practice Research Datalink. CPRD Ethnicity Records. https://www.cprd.com/data/algorithm-derived-data/cprd-ethnicity-records. Accessed 2 October 2025.

[19] Eggermont AMM, Blank CU, Mandala M, Long GV, Atkinson VG, Dalle S, et al. Adjuvant pembrolizumab versus placebo in resected stage III melanoma (EORTC 1325-MG/KEYNOTE-054): distant metastasis-free survival results from a double-blind, randomised, controlled, phase 3 trial. Lancet Oncol. 2021;22:643–54. 33857412 10.1016/S1470-2045(21)00065-6

[20] Davidson, J and Warren-Gash, C (2022). Clinical codelist - CPRD Aurum - learning and intellectual disability. [Data Collection]. London School of Hygiene & Tropical Medicine, London, United Kingdom. 10.17037/DATA.00002809. Accessed 4 December 2024.

[21] Root, A (2019). Clinical Codelist - Intellectual Disability Codes. [Data Collection]. London School of Hygiene & Tropical Medicine, London, United Kingdom. 10.17037/DATA.00001436. Accessed 9 November 2024.

[22] OpenSAFELY. OpenCodelists. Intellectual disability. codelist ID: opensafely/intellectual-disability. 2020. https://www.opencodelists.org/codelist/opensafely/intellectual-disability/2020-08-27. Accessed 30 April 2025.

[23] Kontopantelis E, Olier I, Planner C, Reeves D, Ashcroft DM, Gask L, et al. Primary care consultation rates among people with and without severe mental illness: a UK cohort study using the clinical practice research datalink. BMJ Open. 2015;5:e008650. 26674496 10.1136/bmjopen-2015-008650 PMC4691766

[24] Reilly S, Olier I, Planner C, Doran T, Reeves D, Ashcroft DM, et al. Inequalities in physical comorbidity: a longitudinal comparative cohort study of people with severe mental illness in the UK. BMJ Open. 2015;5:e009010. 26671955 10.1136/bmjopen-2015-009010 PMC4679912

[25] Carey IM, Hosking FJ, Harris T, DeWilde S, Beighton C, Cook DG An evaluation of the effectiveness of annual health checks and quality of health care for adults with intellectual disability: an observational study using a primary care database. Health Services and Delivery Research. Southampton (UK) 2017. 28930382

[26] Harvey PR, Thomas T, Chandan JS, Bhala N, Nirantharakumar K, Trudgill NJ. Outcomes following feeding gastrostomy (FG) insertion in patients with learning disability: a retrospective cohort study using the health improvement network (THIN) database. BMJ Open. 2019;9:e026714. 31221879 10.1136/bmjopen-2018-026714 PMC6588980

[27] OpenSAFELY. OpenCodelists. Learning disabilities. Codelist ID: opensafely/learning-disabilities. https://www.opencodelists.org/codelist/opensafely/learning-disabilities/2020-07-06. Accessed 30th April 2025.

[28] QResearch. QCode group library. Learning disability (LD) codes from QOF excl downs. https://www.qresearch.org/data/qcode-group-library. Accessed 30 April 2025.

[29] Public Health England. Learning disabilities observatory. People with learning disabilities in England 2015: Main report. Version 1.0. November 2016.

[30] Department for Education. Special educational needs information act. An Analysis 2011. https://assets.publishing.service.gov.uk/media/5a7c1419e5274a25a9140514/main_20text_20osr202011.pdf. Accessed 1 May 2025.

[31] Watson J, Nicholson BD, Hamilton W, Price S. Identifying clinical features in primary care electronic health record studies: methods for codelist development. BMJ Open. 2017;7:e019637. 29170293 10.1136/bmjopen-2017-019637 PMC5719324

[32] Sung H, Siegel RL, Laversanne M, Jiang C, Morgan E, Zahwe M, et al. Colorectal cancer incidence trends in younger versus older adults: an analysis of population-based cancer registry data. Lancet Oncol. 2025;26:51–63. 39674189 10.1016/S1470-2045(24)00600-4 PMC11695264

[33] Wang SV, Schneeweiss S. A framework for visualizing study designs and data observability in electronic health record data. Clin Epidemiol. 2022;14:601–8. 35520277 10.2147/CLEP.S358583 PMC9063805

[34] Benchimol EI, Smeeth L, Guttmann A, Harron K, Moher D, Petersen I, et al. The REporting of studies conducted using observational routinely-collected health data (RECORD) statement. PLoS Med. 2015;12:e1001885. 26440803 10.1371/journal.pmed.1001885 PMC4595218

[35] Patel SG, Karlitz JJ, Yen T, Lieu CH, Boland CR. The rising tide of early-onset colorectal cancer: a comprehensive review of epidemiology, clinical features, biology, risk factors, prevention, and early detection. Lancet Gastroenterol Hepatol. 2022;7:262–74. 35090605 10.1016/S2468-1253(21)00426-X

[36] Public Health England. Guidance. Obesity and weight management for people with learning disabilities. 2020.

[37] Public Health England. Guidance. Substance misuse in people with learning disabilities: reasonable adjustments guidance. 2016.

[38] Banda A, Cuypers M, Naaldenberg J, Timen A, Leusink G. Cancer screening participation and outcomes among people with an intellectual disability in the Netherlands: a cross-sectional population-based study. Lancet Public Health. 2025;10:e237–e45. 40044245 10.1016/S2468-2667(25)00011-8

[39] Ward LM, Cooper SA, Sosenko F, Morrison D, Fleming M, McCowan C, et al. Population-based cancer incidence and mortality rates and ratios among adults with intellectual disabilities in Scotland: a retrospective cohort study with record linkage. BMJ Open. 2024;14:e084421. 39142671 10.1136/bmjopen-2024-084421 PMC11331995

[40] Willis D, Samalin E, Satgé D. Colorectal cancer in people with intellectual disabilities. Oncology. 2018;95:323–36. 30173217 10.1159/000492077

[41] Ng AP, Kim S, Chervu N, Gao Z, Mallick S, Benharash P, et al. Disparities in outcomes of colorectal cancer surgery among adults with intellectual and developmental disabilities. PLoS One. 2024;19:e0308938. 39190755 10.1371/journal.pone.0308938 PMC11349222

[42] Mencap. How common is learning disability in the UK? https://www.mencap.org.uk/learning-disability-explained/research-and-statistics/how-common-learning-disability. Accessed 12 Feb 2026.

[43] Learning from Lives and Deaths (LeDeR). Bowel cancer for adults with a learning disability. London: Institute of Psychiatry, Psychology and Neuroscience (IoPPN), King’s College London; 2024. https://www.kcl.ac.uk/ioppn/leder/bowel-cancer-deep-dive.pdf. Accessed 1 March 2026.

