# Supplementary material for: Bowel cancer care in individuals with an intellectual disability: a population-based cohort study of symptoms, diagnostic pathways, treatment and survival
Source: BMC Med. 2026 May 20;24:321. doi: 10.1186/s12916-026-04906-9 (PMC13188521; doi:10.1186/s12916-026-04906-9)
Supplement: Supplementary file 1 — Additional File 1: Supplementary Table 1. A list of codes to identify intellectual disabilities; Supplementary Table 2. A list of codes to identify symptoms testing of potentially indicative of bowel cancer [file 12916_2026_4906_MOESM1_ESM.pdf]

Bowel Cancer Care in Individuals with an Intellectual Disability: A Population-Based Cohort Study of Symptoms, Diagnostic Pathways, Treatment and Survival

Oliver John Kennedy[1,2], Umesh Chauhan[3], Louise Gorman[4], Paul Lorigan[1,2], Samuel W D Merriel[5], Antonia Perumal[6], Tjeerd Van Staa[7], Alison Wright[7,8], Darren Mark Ashcroft[4,7,8]

1. Division of Cancer Sciences, University of Manchester, Manchester, UK
2. The Christie NHS Foundation Trust, Manchester, UK
3. School of Medicine, University of Lancashire, Lancashire, UK
4. NIHR Greater Manchester Patient Safety Research Collaboration, University of Manchester, Manchester, UK
5. Centre for Primary Care and Health Services Research, University of Manchester, Manchester, UK.
6. East Lancashire Hospitals NHS Trust, Royal Blackburn Hospital, Haslingden Road, Blackburn, Lancashire, BB2 3HH
7. Centre for Pharmacoepidemiology and Drug Safety, Division of Pharmacy and Optometry, School of Health Sciences, Faculty of Biology, Medicine and Health, University of Manchester, Manchester, UK.
8. Manchester Academic Health Science Centre, Manchester, UK.

Keywords: learning disability, intellectual disability, bowel cancer; bowel cancer screening

Supplementary Table 1. A list of codes to identify intellectual disabilities.

MedCodeId	OriginalReadCode	CleansedReadCode	Term	SnomedCTConceptId	SnomedCTDescriptionId
2548475019	918e	918e.00	On learning disability register	416075005	2548475019
296557014	Eu70y	Eu70y00	[X]Mild mental retardation, other impairments of behaviour	86765009	143892017
507246016	E30	E30..00	Mild mental retardation, IQ in range 50-70	86765009	507246016
398381000006119	Eu70	Eu70.00	[X]Mild mental retardation	86765009	507246016
398391000006116	Eu70z	Eu70z00	Mild intellectual disability	86765009	3643513017
398411000006116	Eu70-2	Eu70.12	[X]Mild mental subnormality	86765009	507246016
882771000006119	E30-99	E30..99	Mild mental retardation	86765009	882771000006119
1550041000000110	Eu816	Eu81600	Mild learning disability	984661000000105	2504041000000113
2730391000000116	^ESCT1171812		Mild intellectual development disorder without significant impairment of behaviour	1089831000000105	2730391000000116
2730411000000116	^ESCT1171814		Mild intellectual development disorder with significant impairment of behaviour	1089841000000101	2730411000000116
2730431000000112	^ESCT1171816		Mild intellectual development disorder with minimal impairment of behaviour	1089851000000103	2730431000000112
2740421000000114	^ESCT1171813		Mild mental retardation without significant impairment of behaviour	1089831000000105	2740421000000114
2740451000000116	^ESCT1172258		Mild mental retardation with impairment of behaviour	1093991000000101	2740451000000116
2740461000000118	^ESCT1172257		Mild intellectual development disorder with impairment of behaviour	1093991000000101	2740461000000118
3910901000006110	^ESCTMI391090		Mild mental handicap	86765009	507245017
3910931000006119	^ESCTMI391093		Mild learning disability, intelligence quotient in range 50-70	86765009	536871000000113
3910951000006114	^ESCTMI391095		Mild learning disability	86765009	1666311000000112
12202451000006111	^ESCT1220245		Mild intellectual development disorder	86765009	3654174011
12703781000006112	^ESCT1270378		Mild mental retardation (I.Q. 50-70)	86765009	143892017
398811000006118	Eu71	Eu71.00	Moderate intellectual disability	61152003	3643518014
398821000006114	Eu71-1	Eu71.11	[X]Moderate mental subnormality	61152003	3643518014
700071000006118	E310	E310.00	Moderate mental retardation, IQ in range 35-49	61152003	3643518014
882781000006116	E310-99	E310.99	Moderate mental retardation	61152003	882781000006116
1129811000000119	Eu814	Eu81400	Moderate learning disability	984671000000103	2504061000000114
2730291000000112	^ESCT1171804		Moderate intellectual development disorder without significant impairment of behaviour	1089781000000100	2730291000000112
2730311000000113	^ESCT1171806		Moderate intellectual development disorder with significant impairment of behaviour	1089791000000103	2730311000000113
2730351000000112	^ESCT1171808		Moderate intellectual development disorder with minimal impairment of behaviour	1089811000000102	2730351000000112
2730371000000115	^ESCT1171810		Moderate intellectual development disorder with impairment of behaviour	1089821000000108	2730371000000115

3493641000006112	^ESCTMO349364		Moderate mental handicap	61152003	1232179016
3493681000006118	^ESCTMO349368		Moderate learning disability	61152003	1666321000000118
12703791000006110	^ESCT1270379		Moderate mental retardation (I.Q. 35-49)	61152003	101619019
296574014	Eu72y	Eu72y00	[X]Severe mental retardation, other impairments of behaviour	40700009	67882016
146051000006113	E311	E311.00	Severe mental retardation, IQ in range 20-34	40700009	3643515012
201751000006110	E312	E312.00	Profound mental retardation with IQ less than 20	31216003	3643527010
423481000006119	Eu731	Eu73100	[X]Profound ment retard sig impairmnt behav req attent/treat	31216003	52225019
423491000006116	Eu730	Eu73000	[X]Profound ment retrd wth statement no or min impairm behav	31216003	52225019
423501000006112	Eu73	Eu73.00	Profound intellectual disability	31216003	3643527010
423511000006110	Eu73y	Eu73y00	[X]Profound mental retardation, other impairments of behavr	31216003	52225019
423521000006119	Eu73-1	Eu73.11	[X]Profound mental subnormality	31216003	3643527010
426591000006111	Eu72	Eu72.00	Severe intellectual disability	40700009	3643515012
426611000006117	Eu72-1	Eu72.11	[X]Severe mental subnormality	40700009	3643515012
882791000006118	E311-99	E311.99	Severe mental retardation	40700009	882791000006118
1129781000000117	Eu815	Eu81500	Severe learning disability	508171000000105	1129801000000116
1550051000000113	Eu817	Eu81700	Profound learning disability	984681000000101	2504081000000117
2730131000000115	^ESCT1171788		Profound intellectual development disorder without impairment of behaviour	1089701000000105	2730131000000115
2730191000000119	^ESCT1171794		Profound intellectual development disorder with impairment of behaviour	1089731000000104	2730191000000119
2730211000000115	^ESCT1171796		Severe intellectual development disorder without significant impairment of behaviour	1089741000000108	2730211000000115
2730231000000111	^ESCT1171798		Severe intellectual development disorder with significant impairment of behaviour	1089751000000106	2730231000000111
2730251000000116	^ESCT1171800		Severe intellectual development disorder with minimal impairment of behaviour	1089761000000109	2730251000000116
2730271000000113	^ESCT1171802		Severe intellectual development disorder with impairment of behaviour	1089771000000102	2730271000000113
2740341000000113	^ESCT1171797		Severe mental retardation without significant impairment of behaviour	1089741000000108	2740341000000113
3003301000006113	^ESCTPR300330		Profound mental handicap	31216003	1227448012
3003361000006114	^ESCTPR300336		Profound learning disability	31216003	1666271000000112
3155151000006112	^ESCTSE315515		Severe learning disability	40700009	2310251000000113
3155171000006119	^ESCTSE315517		Severe mental handicap	40700009	1229669012
12009301000006116	^ESCT1200930		Hypotonia, speech impairment, severe cognitive delay syndrome	763722004	3643495013
12177541000006119	^ESCT1217754		Profound intellectual development disorder	31216003	3654215011
12703921000006110	^ESCT1270392		Profound mental retardation (I.Q. below 20)	31216003	52225019
12703941000006115	^ESCT1270394		Severe mental retardation (I.Q. 20-34)	40700009	67882016
13622001000006116	^ESCT1362200		Severe intellectual disability, progressive spastic diplegia syndrome	782723007	3755494018

15004801000006113	^ESCT1500480		Congenital insensitivity to pain with severe intellectual disability	1237623009	5100092010
378493013	PJ0z	PJ0z.00	Down's syndrome NOS	41040004	598021000000114
378494019	PJ02-1	PJ02.11	Partial trisomy 21 in Down's syndrome	254264002	378494019
1224878018	PJ0z-1	PJ0z.11	Trisomy 21 NOS	41040004	222121000000113
3528203016	^ESCT1167827		Trisomy 21	737542000	3528203016
88351000006114	PJ00	PJ00.00	Trisomy 21, meiotic nondisjunction	205615000	315346017
88361000006111	PJ01-1	PJ01.11	Trisomy 21, mitotic nondisjunction	205616004	315347014
88371000006116	PJ01	PJ01.00	Trisomy 21- mitotic nondisjunction mosaicism	205616004	315347014
88381000006118	PJ02	PJ02.00	Trisomy 21, translocation	254264002	378495018
222121000000113	PJ0-2	PJ0..12	Trisomy 21	41040004	68470016
628281000006114	PJ0	PJ0..00	Down's syndrome	41040004	598021000000114
893481000006117	PJ0-98	PJ0..98	Down's syndrome	41040004	893481000006117
1009531000006118	EMISNQCA42		Cause of learning disabilities: Down's syndrome	1009531000006102	1009531000006118
3161031000006112	^ESCTCO316103		Complete trisomy 21 syndrome	41040004	68470016
3161041000006119	^ESCTDO316104		Down syndrome	41040004	68471017
3161051000006117	^ESCTT2316105		T21 - Trisomy 21	41040004	1229711010
3161061000006115	^ESCTDO316106		Downs syndrome	41040004	2921053011
4830941000006112	^ESCTTR483094		Trisomy 21- meiotic nondisjunction	205615000	315346017
6348371000006113	^ESCTTR634837		Translocation Down syndrome	371045000	1209755010
7955161000006118	^ESCTDE795516		Dementia with Down syndrome	733194007	3498962017
8039101000006116	^ESCTFE803910		Fetal trisomy 21, Down syndrome	125501000119105	3004785019
13944201000006118	^ESCT1394420		Down syndrome co-occurrent with leukaemoid reaction associated transient neonatal pustulosis	840505007	3902179014
2090010	PJyy4	PJyy400	Fragile X syndrome	613003	2090010
4159010	P21	P21..00	Microcephalus	1829003	4159010
4161018	P211	P211.00	Micrencephaly	1829003	4161018
5179014	P02	P02..00	Iniencephaly	2438005	5179014
9538013	P2280-1	P228011	Agenesis of corpus callosum	5102002	9538013
10374011	PKy1	PKy1.00	Laurence-Moon-Biedl syndrome	5619004	10374011
12877016	PK5	PK5..00	Tuberous sclerosis	7199000	12877016
17479015	ESCTCO12		Coffin-Siris syndrome	10007009	17479015
18114013	C3723	C372300	Lesch-Nyhan syndrome	10406007	18114013
19694018	C375	C375.00	Mucopolysaccharidosis	11380006	19694018
25344018	PKy65	PKy6500	Aarskog syndrome	14921002	25344018
25776014	PKy5F	PKy5F00	Coffin-Lowry syndrome	15182000	25776014
33473011	PK61	PK61.00	Sturge-Weber syndrome	19886006	33473011
35440018	PKy61	PKy6100	Cockayne syndrome	21086008	35440018
36300015	PKy69	PKy6900	Borjeson-Forssman-Lehmann syndrome	21634003	36300015
40055016	F1016	F101600	Sandhoff disease	23849003	40055016
49116010	PG442-1	PG44211	Thanatophoric dysplasia	29352008	49116010
51766016	P225	P225.00	Holoprosencephaly	30915001	51766016
51767013	P224	P224.00	Arhinencephaly	30915001	51767013

53817017	P01	P01..00	Craniorachischisis	32219008	53817017
55063010	PJ335	PJ33500	Greig cephalopolysyndactyly syndrome	32985001	55063010
58597010	PJy2	PJy2.00	XXX syndrome	35111009	1228249015
63896011	PKy60-1	PKy6011	Cornelia de Lange syndrome	40354009	63896011
63898012	PKy60	PKy6000	Amsterdam dwarf	40354009	63898012
68472012	PJ0-1	PJ0..11	Mongolism	41040004	598021000000114
68519010	PJ339	PJ33900	Langer-Giedion syndrome	41069008	68519010
76004014	PKy73-1	PKy7311	Rubinstein-Taybi syndrome	45582004	76004014
81676016	PJ513	PJ51300	Trisomy 4p syndrome	49024004	81676016
94131019	PKy5K	PKy5K00	Cohen syndrome	56604005	94131019
96285014	PKy64	PKy6400	Seckel syndrome	57917004	96285014
97154016	C3272	C327200	Niemann-Pick disease	58459009	97154016
104922018	PKy03	PKy0300	Weaver syndrome	63119004	104922018
108539019	C375-4	C375.14	Lipochondrodystrophy	378007	486814011
108540017	C3751-1	C375111	Gargoylism	75610003	503067012
108541018	C3751-2	C375112	Hurler's syndrome	65327002	108541018
110901011	PG5F	PG5F.00	Acrodysostosis	66758006	110901011
116814019	PJ515	PJ51500	15q partial trisomy syndrome	70324008	116814019
119577010	E141-1	E141.11	Heller's syndrome	71961003	119577010
119579013	E141	E141.00	Disintegrative psychosis	71961003	119579013
121311016	PJ512	PJ51200	10q partial trisomy syndrome	73035005	121311016
121446013	C3751-3	C375113	Scheie's syndrome	73123008	1233590012
121704017	PKy04	PKy0400	Marshall-Smith syndrome	73284007	121704017
126200014	PKyM	PKyM.00	Johanson-Blizzard syndrome	75979009	126200014
127638013	PKyz5-1	PKyz511	Angelman syndrome	76880004	1234038018
127639017	PKyz5	PKyz500	Happy puppet syndrome	76880004	127639017
128696013	PJ514	PJ51400	Trisomy 9p syndrome	77527000	128696013
131704013	C3025-1	C302511	Oculocerebrorenal syndrome	79385002	131704013
137830014	PJ3y0	PJ3y000	Shprintzen syndrome	767263007	3670123013
148214012	PKy0-2	PKy0.12	Prader-Willi syndrome	89392001	148214012
148320010	PJ535	PJ53500	Shwachman-Diamond syndrome	89454001	148320010
151009017	E3	E3...00	Mental retardation	110359009	3643707012
187765019	F101-1	F101.11	Amaurotic familial idiocy	61663001	102454019
215930017	9F8	9F8..00	Statement of special educational needs	134188003	215930017
251223010	13Z4E	13Z4E00	Learning difficulties	161129001	251223010
264621015	6664	6664.00	Mental handicap problem	170695009	264621015
293559017	C372z	C372z00	Other disorder of purine or pyrimidine metabolism NOS	238006008	356736012
293603019	C375z	C375z00	Mucopolysaccharidosis NOS	11380006	19694018
294938012	E1410	E141000	Active disintegrative psychoses	191692007	294938012
294939016	E1411	E141100	Residual disintegrative psychoses	191693002	294939016
294941015	E141z	E141z00	Disintegrative psychosis NOS	71961003	119576018
295622013	E2E1	E2E1.00	Hyperkinesis with developmental delay	192131001	295622013
295633011	E2F2	E2F2.00	Specific learning difficulty	161129001	251223010

295642016	E2F3z	E2F3z00	Speech or language developmental disorder NOS	268672004	401825015
295651012	E2Fy	E2Fy.00	Developmental disorder	5294002	9881017
295652017	E2Fz	E2Fz.00	Developmental disorder NOS	5294002	9881017
295661017	E3y	E3y..00	Other specified mental retardation	110359009	175156010
295662012	E31z	E31z.00	Other specified mental retardation NOS	110359009	175156010
295664013	E3z	E3z..00	Intellectual disability	110359009	175156010
296565012	Eu71y	Eu71y00	[X]Mod retard oth behav impair	61152003	101619019
296586012	Eu7y	Eu7y.00	[X]Other mental retardation	110359009	175156010
296592018	Eu7yy	Eu7yy00	[X]Other mental retardation, other impairments of behaviour	110359009	175156010
296643017	Eu81	Eu81.00	[X]Specific developmental disorders of scholastic skills	1855002	4203013
296657015	Eu81z	Eu81z00	Developmental disorder of scholastic skill	1855002	478661012
296663012	Eu83	Eu83.00	Mixed developmental disorder	442059001	2819191012
296683013	Eu8z	Eu8z.00	Disorder of psychological development	192562009	296607011
296991018	F1021	F102100	Cerebral degeneration in Niemann-Pick disease	192792002	296991018
296995010	F1031	F103100	Cerebral degeneration in mucopolysaccharidoses	192796004	296995010
312368018	E2F	E2F..00	Specific delays in development	10720004	312368018
312877018	P21z	P21z.00	Microcephalus NOS	1829003	4159010
312878011	P22	P22..00	Reduction deformities of brain	204032005	312878011
312902012	P22y	P22y.00	Other specified reduction deformities of brain	204032005	312878011
312905014	P22yz	P22yz00	Other reduction deformity of brain NOS	204032005	312878011
314772015	PF550	PF55000	Acrocephalosyndactyly (Apert)	205258009	314772015
314773013	PF551	PF55100	Acrocephalosyndactyly type V	70410008	2816530018
315143015	PG5D	PG5D.00	Craniodiaphyseal dysplasia	205506004	315143015
315350012	PJ10	PJ10.00	Trisomy 13, meiotic nondisjunction	205619006	315350012
315359013	PJ3	PJ3..00	Monosomies and deletions from the autosomes	205627002	315359013
315360015	PJ30	PJ30.00	Antimongolism syndrome	254274004	378508010
315361016	PJ33	PJ33.00	Other deletions of part of a chromosome	254274004	378508010
315362011	PJ330	PJ33000	Deletion of long arm of chromosome 13	205630009	315362011
315369019	PJ33z	PJ33z00	Other deletion of part of a chromosome NOS	254274004	378508010
315371019	PJ35	PJ35.00	Deletions with other complex rearrangements	274908005	410807013
315377015	PJ370	PJ37000	Monosomy 21, mosaicism	205638002	315377015
315378013	PJ37z	PJ37z00	Whole chromosome monosomy, mosaicism NOS	270520003	405158019
315383017	PJ3y	PJ3y.00	Other deletions from the autosomes	254274004	378508010
315385012	PJ3z	PJ3z.00	Monosomies and deletions from the autosomes NOS	254274004	378508010
315387016	PJ5	PJ5..00	Other condition due to autosomal anomaly	74345006	123461015
315389018	PJ500	PJ50000	Trisomy 6	205647005	315389018
315390010	PJ501	PJ50100	Trisomy 7	205648000	315390010
315391014	PJ502	PJ50200	Trisomy 8	205649008	315391014
315392019	PJ503	PJ50300	Trisomy 9	205650008	315392019
315393012	PJ504	PJ50400	Trisomy 10	205651007	315393012
315394018	PJ505	PJ50500	Trisomy 11	205652000	315394018
315395017	PJ506	PJ50600	Trisomy 12	205653005	315395017
315396016	PJ507	PJ50700	Other trisomy C syndromes	270521004	405160017

315397013	PJ508	PJ50800	Trisomy 22	205655003	315397013
315398015	PJ50w	PJ50w00	Whole chromosome trisomy meiotic nondisjunction	254269007	3661470014
315399011	PJ50x	PJ50x00	Whole chromosome trisomy, mosaicism	205657006	315399011
315401017	PJ50y	PJ50y00	Other specified whole chromosome trisomy syndrome	270521004	405160017
315402012	PJ50z	PJ50z00	Whole chromosome trisomy syndrome NOS	270521004	405160017
315403019	PJ51	PJ51.00	Partial trisomy syndromes	205660004	315403019
315404013	PJ510	PJ51000	Major partial trisomy	205661000	315404013
315405014	PJ511	PJ51100	Minor partial trisomy	205662007	315405014
315406010	PJ51z	PJ51z00	Partial trisomy syndrome NOS	205660004	315403019
315407018	PJ52	PJ52.00	Trisomies of autosomes NEC	270521004	405160017
315410013	PJ522	PJ52200	Extra marker chromosomes	444655009	2870837017
315413010	PJ52z	PJ52z00	Trisomy and partial trisomy of autosome	270521004	405160017
315418018	PJ531	PJ53100	Balanced autosomal rearrangement in abnormal individual	205673000	315418018
315419014	PJ532	PJ53200	Balanced sex/autosomal rearrangement in abnormal individual	205674006	315419014
315420015	PJ533	PJ53300	Individual with marker heterochromatin	205675007	315420015
315421016	PJ534	PJ53400	Individual with autosomal fragile site	205676008	315421016
315474014	PJy13	PJy1300	Mosaic including XXXXY	205710004	315474014
315486012	PJyy2	PJyy200	Fragile X chromosome	205720009	315486012
315493011	PJz2	PJz2.00	Deletion of part of autosome	254274004	378508010
315571015	Q0071-1	Q007111	Fetal alcohol syndrome	205788004	315571015
315607013	PKy70	PKy7000	Carpenter syndrome	403767009	3494870015
315625018	PKy80	PKy8000	Noonan's syndrome	205824006	315625018
315655013	Pyu02	Pyu0200	[X]Other reduction deformities of brain	204032005	312878011
315656014	Pyu03	Pyu0300	[X]Other specified congenital malformations of brain	88425004	146609011
315799014	PyuA1	PyuA100	[X]Other deletions of part of a chromosome	254274004	378508010
315800013	PyuA2	PyuA200	[X]Other deletions from the autosomes	205627002	315358017
329968011	C03z-2	C03z.12	Cretinism	217710005	329968011
342159015	13ZK	13ZK.00	Child with special educational needs	228141003	342159015
342177013	13VCA		Intellectual functioning disability	228156007	342177013
345138018	F1306	F130600	Aicardi Goutieres syndrome	230312006	345138018
345281011	F2505	F250500	Lennox-Gastaut syndrome	230418006	345281011
347021018	E2F3-1	E2F3.11	Developmental language disorder	280032002	417527019
377050018	P2402	P240200	Schizencephaly	253159001	377050018
377071011	P22y2	P22y200	Gillespie syndrome	253176002	377071011
378221016	C375-A	C375.1A	Dysostosis multiplex	254069004	378221016
378490011	PJ5z-1	PJ5z.11	Aneuploidy NEC	74345006	200444011
378491010	PJ5y-1	PJ5y.11	Pseudotrisomy 18	254261005	378491010
378496017	PJ2z	PJ2z.00	Edward's syndrome NOS	51500006	85775018
378497014	PJ22-1	PJ22.11	Partial trisomy 18 in Edward's syndrome	254266000	378497014
378499012	PJ1z	PJ1z.00	Patau's syndrome NOS	21111006	35482019
378500015	PJ12-1	PJ12.11	Partial trisomy 13 in Patau's syndrome	254268004	378500015
378507017	PJ37-2	PJ37.12	Autosomal deletion - mosaicism	254273005	378507017
398888012	C372	C372.00	Disorder of purine and pyrimidine metabolism	238006008	356736012

400767012	P22z	P22z.00	Reduction deformities of brain NOS	204032005	312878011
400910012	PF55	PF55.00	Acrocephalosyndactyly	268262006	400910012
400911011	PF55-1	PF55.11	Apert's syndrome	268262006	400911011
400957013	PJ38	PJ38.00	Chromosome replaced with ring or dicentric	268294001	400957013
400959011	PJ5y	PJ5y.00	Other specified conditions due to autosomal anomalies	74345006	123461015
400960018	PJ5z	PJ5z.00	Unspecified conditions due to autosomal anomalies	74345006	123461015
400966012	PKy0	PKy0.00	Multiple system congenital anomalies NEC	66091009	109766011
401827011	E2F30	E2F3000	Developmental aphasia	268673009	401827011
401902015	Eu7z	Eu7z.00	[X]Unspecified mental retardation	110359009	175156010
401906017	Eu80y	Eu80y00	[X]Other developmental disorders of speech and language	268672004	401825015
401909012	Eu81y	Eu81y00	[X]Other developmental disorders of scholastic skills	1855002	4203013
401910019	Eu843	Eu84300	[X]Other childhood disintegrative disorder	35919005	59939011
401911015	Eu8y	Eu8y.00	[X]Other disorders of psychological development	5294002	9881017
405159010	PJ37	PJ37.00	Whole chromosome monosomy, mosaicism	270520003	405159010
405346013	PJ331	PJ33100	Deletion of long arm of chromosome 18	270889005	405346013
405347016	PJ332-1	PJ33211	18q- syndrome	270889005	405347016
405348014	PJ331-1	PJ33111	18p- syndrome	270890001	405348014
405349018	PJ332	PJ33200	Deletion of short arm of chromosome 18	270890001	405349018
413177014	E30-1	E30..11	Educationally subnormal	276854003	413177014
415431010	C031	C031.00	Goitrous cretin	278503003	415431010
455888019	PKy68	PKy6800	Floating-Harbor syndrome	312214005	455888019
456373012	8O07	8O07.00	Provision of special educational needs nursery	312621002	456373012
460157017	8O5	8O5..00	Special needs support	315638005	460157017
460622019	ZV400	ZV40000	[V]Problems with learning	161129001	251223010
497080018	C3758	C375800	Multiple sulphatase deficiency	54898003	497080018
500490013	PJ523	PJ52300	Triploidy	66651005	500490013
502310015	PJ524	PJ52400	Polyploidy	72991005	502310015
502891013	C0A	C0A..00	Congenital iodine deficiency syndrome	75065003	502891013
1221474011	PJ32	PJ32.00	Deletion of short arm of chromosome 4	17122004	1221474011
1222495016	R034y-1	R034y11	[D]Global retardation	224958001	338115019
1224879014	PJ2z-1	PJ2z.11	Trisomy 18 NOS	51500006	85775018
1224880012	PJ1z-1	PJ1z.11	Trisomy 13 NOS	21111006	35482019
1224941015	PKy66	PKy6600	Dubowitz syndrome	2593002	1224941015
1226349010	PG442	PG44200	Thanatophoric dwarfism	29352008	1226349010
1228248011	PJy2-1	PJy2.11	Triple X female	35111009	1228248011
1228249015	PJy2-2	PJy2.12	Karyotype 47, XXX	35111009	1228249015
1229637015	PKy60-3	PKy6013	Degenerative amsterodamensis typus	40354009	1229637015
1229639017	PKy60-2	PKy6012	Bruck-de Lange syndrome	40354009	1229639017
1231577014	PKy1-1	PKy1.11	Biedl-Bardet syndrome	5619004	1231577014
1232445010	PKy4	PKy4.00	William syndrome	63247009	1232445010
1232670018	C3082	C308200	X-linked adrenoleucodystrophy	65389002	1232670018
1233229018	PJ31-1	PJ31.11	Deletion of short arm of chromosome 5	70173007	1233229018
1233939016	C1zy2	C1zy200	Cerebral gigantism	75968004	1233939016

1233940019	C1zy2-1	C1zy211	Sotos syndrome	75968004	1233940019
1234038018	PKyz7	PKyz700	Angelman's syndrome	76880004	1234038018
1234349019	C3025-2	C302512	Oculocerebrorenal dystrophy	79385002	1234349019
1234352010	C3025	C302500	Lowe disease	79385002	1234352010
1234503010	P2283	P228300	Aicardi syndrome	80651009	1234503010
1234786015	PJ3y0-1	PJ3y011	Velocardiofacial syndrome	767263007	3670117015
1235347013	PKy94	PKy9400	Zellweger's syndrome	88469006	1235347013
1235773018	B927-2	B927.12	Neurofibromatosis type 1	92824003	1235773018
1488521019	9bA0	9bA0.00	Mental handicap (specialty)	394815005	1488521019
1491711019	Eu800-2	Eu80012	[X]Developmental speech articulation disorder	386701004	1491709011
1494665012	PyuAB	PyuAB00	Pallister-Killian syndrome	9527009	3010792013
1780502018	PJ333	PJ33300	Smith-Magenis syndrome	401315004	1780502018
1786608015	1P01	1P01.00	Psychomotor retardation	398991009	1786608015
2159902018	1JB0	1JB0.00	Suspected Downs syndrome	408338009	2159902018
2478440016	PKyG-1	PKyG.11	Ohdo blepharophimosis syndrome	412787009	2478440016
2900005013	F1y0	F1y0.00	Fragile X associated tremor ataxia syndrome	448045004	2900005013
2995031014	PKy06	PKy0600	Feingold syndrome	702431004	2995031014
3505252012	^ESCT1164321		Alpha-thalassaemia intellectual disability syndrome linked to chromosome 16	734349003	3505252012
3636386011	^ESCT1168995		Developmental language disorder and impairment of receptive and expressive language	762317007	3636386011
3636387019	^ESCT1168996		Developmental language disorder co-occurrent with impairment of receptive and expressive language	762317007	3636387019
3636390013	^ESCT1168997		Developmental language disorder and impairment of expressive language	762318002	3636390013
3636779018	^ESCT1169234		Developmental language disorder co-occurrent with language impairment	762502009	3636779018
3636780015	^ESCT1169233		Developmental language disorder and language impairment	762502009	3636780015
9881000006115	E31	E31..00	Other specified mental retardation	110359009	175156010
53891000006113	PJ32-1	PJ32.11	Wolff - Hirschorn syndrome	17122004	1221473017
60491000006112	PJ36	PJ36.00	Whole chromosome monosomy, meiotic nondisjunction	205636003	315372014
60541000006119	PJ50	PJ50.00	Whole chromosome trisomy syndromes	205646001	315388014
63811000006114	B927-1	B927.11	Von Recklinghausen's disease	92824003	1235773018
79051000006114	PKyz0	PKyz000	Ullrich - Feichtiger syndrome, chimaera	21111006	35482019
88251000006115	PJ11-1	PJ11.11	Trisomy 13, mitotic nondisjunction	205620000	315351011
88261000006118	PJ11	PJ11.00	Trisomy 13 - mitotic nondisjunction mosaicism	205620000	315351011
88271000006113	PJ12	PJ12.00	Trisomy 13, translocation	254268004	378501016
88291000006114	PJ20	PJ20.00	Trisomy 18, meiotic nondisjunction	205623003	315354015
88301000006110	PJ21-1	PJ21.11	Trisomy 18, mitotic nondisjunction	205624009	315355019
88311000006113	PJ21	PJ21.00	Trisomy 18 - mitotic nondisjunction mosaicism	205624009	315355019
88321000006117	PJ22	PJ22.00	Trisomy 18, translocation	254266000	378498016
88391000006115	PJ0-3	PJ0..13	Trisomy 22	205655003	315397013
107661000006110	F1013	F101300	Tay-Sach's disease	192787004	296986019

133861000006111	E2F3-2	E2F3.12	Developmental speech disorder	1145003	2999013
133891000006115	E2F3	E2F3.00	Disorder of speech and language development	268672004	401825015
137341000006114	PKy63	PKy6300	Smith - Lemli - Opitz syndrome	43929004	73253014
154901000006117	C375-9	C375.19	Scheie's syndrome	73123008	1233590012
158401000006118	PKy73	PKy7300	Rubenstein - Tayi syndrome	45582004	76004014
212911000006116	PKy93	PKy9300	Prader-Willi syndrome	89392001	148214012
212921000006112	PKy0-1	PKy0.11	Prader-Willi Syndrome	89392001	148214012
215821000000119	E30-3	E30..13	Moron	86765009	507246016
222131000000110	PJ71-1	PJ71.11	Klinefelter's syndrome, XXXY	275263003	411223014
222141000000118	PJ71-2	PJ71.12	Klinefelter's syndrome, XXXXY	275264009	411224015
223441000000119	C3720-1	C372011	Lesch - Nyhan syndrome	10406007	18114013
239991000006111	PJ1	PJ1..00	Patau syndrome	21111006	35483012
253231000006116	F2511-1	F251111	Otohara syndrome	192990004	297280011
297751000006119	PKy0-3	PKy0.13	Noonan's syndrome	205824006	315625018
301521000000111	Eu804	Eu80400	[X]Cocktail party syndrome	268672004	401825015
302051000000118	PKyJ	PKyJ.00	Lujan-Fryns syndrome	422437002	2649528010
362161000006110	Eu803	Eu80300	Acquired epileptic aphasia	230438007	345312010
371571000006114	Eu802-1	Eu80211	[X]Congenital auditory imperception	229748008	344387017
376631000006115	Eu843-1	Eu84311	[X]Dementia infantalis	71961003	119576018
376831000006116	Eu8y-1	Eu8y.11	[X]Developmental agnosia	5294002	9881017
376841000006114	Eu801-2	Eu80112	[X]Developmental aphasia, expressive type	268734000	401904019
376851000006111	Eu802-5	Eu80215	Receptive language delay	229736005	344370019
376891000006117	Eu80z	Eu80z00	[X]Developmental disorder of speech and language unspecified	268672004	401825015
376911000006115	Eu801-1	Eu80111	Developmental expressive language disorder	268734000	401904019
376921000006111	Eu802-2	Eu80212	[X]Developmental dysphasia, receptive type	268673009	401827011
376951000006119	Eu812-3	Eu81213	[X]Developmental Gerstmann's syndrome	229676007	344282013
376961000006117	Eu800-1	Eu80011	Developmental speech articulation disorder	386701004	1491709011
376981000006110	Eu802-3	Eu80213	[X]Developmental Wernicke's aphasia	367515004	492553013
377461000006113	Eu843-2	Eu84312	[X]Disintegrative psychosis	35919005	59939011
377921000006114	Eu8	Eu8..00	[X]Disorders of psychological development	192562009	296607011
386751000006111	Eu70-1	Eu70.11	[X]Feeble-mindedness	86765009	507246016
388761000006114	Eu843-3	Eu84313	[X]Heller's syndrome	71961003	119577010
394581000006118	Eu840-4	Eu84014	[X]Kanner's syndrome	408856003	2477201014
394991000006113	Eu80z-1	Eu80z11	[X]Language development disorder NOS	268672004	401825015
395051000006119	Eu81z-3	Eu81z13	[X]Learn acquisition disab NOS	110359009	3643707012
395061000006117	Eu81z-1	Eu81z11	Learning disability	1855002	478664016
395071000006112	Eu81z-2	Eu81z12	[X]Learning disorder NOS	1855002	478661012
398201000006115	Eu7z-1	Eu7z.11	[X]Mental deficiency NOS	110359009	175156010
398231000006111	Eu7	Eu7..00	[X]Mental retardation	110359009	3643707012
398241000006118	Eu841-2	Eu84112	[X]Mental retardation with autistic features	231536004	347022013
398251000006116	Eu7z-2	Eu7z.12	[X]Mental subnormality NOS	110359009	175156010
398591000006113	Eu813	Eu81300	Mixed disorder of scholastic skills	192575009	296652014

398651000006118	Eu701	Eu70100	[X]Mld mental retard sig impairment behav req attent/treatmt	86765009	143892017
398661000006116	Eu700	Eu70000	[X]Mld mental retard with statement no or min impairm behav	86765009	143892017
398761000006113	Eu711	Eu71100	[X]Mod mental retard sig impairment behav req attent/treatmt	61152003	101619019
398771000006118	Eu710	Eu71000	[X]Mod mental retard with statement no or min impairm behav	61152003	101619019
398781000006115	Eu71z	Eu71z00	[X]Mod mental retardation without mention impairment behav	61152003	3643518014
404851000006117	Eu7y1	Eu7y100	[X]Oth mental retard sig impairment behav req attent/treatmt	110359009	175156010
404861000006115	Eu7y0	Eu7y000	[X]Oth mental retard with statement no or min impairm behav	110359009	175156010
405381000006115	PyuA0	PyuA000	[X]Oth specif trisomies & partial trisomies of autosomes	270521004	405160017
411791000006118	Eu7yz	Eu7yz00	[X]Other mental retardation without mention impairment behav	110359009	175156010
417681000006116	Eu844	Eu84400	[X]Overactive disorder assoc mental retard/stereotype movts	35919005	59939011
423361000006118	Eu73z	Eu73z00	[X]Prfnd mental retardation without mention impairment behav	31216003	52225019
424181000006111	Eu8z-1	Eu8z.11	[X]Psychological developmental disorder NOS	192562009	296607011
424971000006111	Eu842	Eu84200	Rett syndrome	68618008	113977013
426521000006114	Eu721	Eu72100	[X]Sev mental retard sig impairment behav req attent/treatmt	40700009	67882016
426531000006112	Eu720	Eu72000	[X]Sev mental retard with statement no or min impairm behav	40700009	67882016
426541000006119	Eu72z	Eu72z00	[X]Sev mental retardation without mention impairment behav	40700009	3643515012
427261000006119	Eu80	Eu80.00	[X]Specific developmental disorders of speech and language	268672004	401825015
430061000006113	Eu7z0	Eu7z000	[X]Unsp mental retard with statement no or min impairm behav	110359009	175156010
430071000006118	Eu7zz	Eu7zz00	[X]Unsp mental retardation without mention impairment behav	110359009	175156010
430081000006115	Eu7z1	Eu7z100	[X]Unsp mentl retard sig impairment behav req attent/treatmt	110359009	175156010
431231000006115	Eu7zy	Eu7zy00	[X]Unspecified mental retardatn, other impairments of behav	110359009	175156010
539561000006115	PG421-1	PG42111	Catel-Schwartz-Jampel syndrome	29145002	48771010
553151000006114	PJ38-1	PJ38.11	Chromosome replaced with dicentric	268294001	400957013
553161000006111	PJ38-2	PJ38.12	Chromosome replaced with ring	268294001	400957013
557191000000119	PJ9	PJ9..00	Mowat-Wilson syndrome	703535000	3009119013
571541000006116	PKyz-1	PKyz.11	Cockayne's syndrome	21086008	35440018
583291000006115	C0A1	C0A1.00	Congenital iodine-deficiency syndrome, myxoedematous type	75065003	502897012
583301000006119	C0A0	C0A0.00	Congenital iodine-deficiency syndrome, neurological type	237566004	356024011
596611000000111	R034A	R034A00	[D]Communication skills development delay	274625009	410457011
600251000000118	PJ334	PJ33400	Jacobsen syndrome	715438008	3302630014
600791000006112	C03-1	C03..11	Cretinism	217710005	329968011
600811000006111	PJ31	PJ31.00	Cri du chat syndrome	70173007	116547017
610871000006117	C3770	C377000	Defects in post-translational modif'n of lysosomal enzymes	190948002	293609015
612441000006114	PJ30-1	PJ30.11	Deletion of long arm of chromosome 21	254274004	378508010
612481000006115	PJ34	PJ34.00	Deletions seen only at prometaphase	205634000	315370018
616061000006115	C3911	C391100	DiGeorge syndrome	767263007	3670122015
623961000006117	C375X	C375X00	Disorder of glucosaminoglycan metabolism, unspecified	238043005	356812017
623971000006112	C31yX	C31yX00	Disorder of glycoprotein metabolism, unspecified	238045003	356821016
624501000006112	C377	C377.00	Disorder of glycoprotein metabolism	238045003	356821016
632481000006113	PJ520	PJ52000	Duplications seen only at prometaphase	205665009	315408011

632491000006111	PJ521	PJ52100	Duplications with other complex rearrangements	205666005	315409015
636711000006113	PJ2	PJ2..00	Edward's syndrome - trisomy 18	51500006	85776017
667621000006119	E30-2	E30..12	Feeble-minded	86765009	507246016
669211000006118	PK80	PK80.00	Fetal alcohol syndrome	205788004	315571015
676511000006115	B927	B927.00	Neurofibromatosis - Von Recklinghausen's disease	92824003	1235773018
682861000006114	PG421	PG42100	Schwartz-Jampel syndrome	29145002	48771010
696681000006115	C3751	C375100	Mucopolysaccharidosis, type 1	75610003	503067012
696691000006117	C3752	C375200	Mucopolysaccharidosis type II	70737009	501652016
696701000006117	C3753	C375300	Mucopolysaccharidosis type III	88393000	1235342019
696711000006119	C3754	C375400	Mucopolysaccharidosis type IV	378007	486815012
696721000006110	C3756	C375600	Mucopolysaccharidosis, type VI	69463008	501281010
696731000006113	C3757	C375700	Mucopolysaccharidosis type VII	43916004	493711018
698811000006110	PKy92	PKy9200	Menkes syndrome	59178007	198877015
701121000006111	E2F5	E2F5.00	Mixed disorder of psychological development	192147004	295650013
733951000006111	13Z3	13Z3.00	Intelligence quotient low	102942005	256780013
747231000006115	C372-1	C372.11	Lesch - Nyhan syndrome	10406007	18114013
754291000006111	PKy92-1	PKy9211	Kinky hair syndrome	59178007	498370014
785941000006115	E310-1	E310.11	Imbecile	61152003	3643518014
787151000006113	E312-1	E312.11	Idiocy	31216003	3643527010
787441000006118	C3720	C372000	Hypoxanthine-guanine-phosphoribosyltransferase deficiency	124275001	203921018
798681000006113	C375-1	C375.11	Gargoylism	75610003	503067012
803251000006114	E2F5-1	E2F5.11	Global developmental delay	224958001	338115019
829441000006116	C375-3	C375.13	Hurler's syndrome	65327002	108541018
831231000006112	EMISQPS1		Psychomotor retardation	831231000006108	831231000006112
855571000006112	EGTONCH5		Child language development delayed	855571000006108	855571000006112
855591000006113	EGTONCH6		Child language development abnormal	855591000006109	855591000006113
881231000006117	B927-99	B927.99	Neurofibromatosis	92824003	881231000006117
882741000006110	E2Fz-99	E2Fz.99	Development delay - NOS	686491000000105	882741000006110
882761000006114	E3-99	E3...99	Mental subnormality	91138005	882761000006114
882801000006117	E31z-99	E31z.99	Mental subnormality NOS	686511000000102	882801000006117
893501000006110	PJyy2-99	PJyy299	Fragile X syndrome	205720009	893501000006110
905641000006118	HNG0038		[RFC] Development delay	905641000006102	905641000006118
906941000006119	HNG0150		[RFC] Learning disabilities	906941000006103	906941000006119
907741000006115	HNG0250		[RFC] Specific learning problems	907741000006104	907741000006115
908591000006117	HNG0517		[RFC] General development delay	908591000006101	908591000006117
908941000006118	HNG0625		[RFC] Learning disability	908941000006102	908941000006118
909931000006115	HNGNQRF38		[RFC] Special needs	909931000006104	909931000006115
909941000006113	HNGNQRF39		[RFC] Communication special needs	909941000006109	909941000006113
909951000006110	HNGNQRF40		[RFC] Learning special needs	909951000006106	909951000006110
923881000006119	PCNQGE2		General development delay	923881000006103	923881000006119
940371000006118	EMISNQFR2		Fragile X syndrome	940371000006102	940371000006118
968201000006114	PKyG	PKyG.00	Mental retardation, congenital heart disease, blepharophimosis, blepharoptosis and hypoplastic teeth	412787009	2474343011

988941000006119	E3-98	E3...98	Mental subnormality NOS	91138005	988941000006119
1009521000006116	EMISNQCA41		Cause of learning disabilities	1009521000006100	1009521000006116
1009541000006111	EMISNQCA43		Cause of learning disabilities: Tuberous sclerosis	1009541000006107	1009541000006111
1009551000006113	EMISNQCA44		Cause of learning disabilities: Birth trauma	1009551000006109	1009551000006113
1009561000006110	EMISNQCA45		Cause of learning disabilities: Meningitis/encephalitis	1009561000006106	1009561000006110
1009571000006115	EMISNQCA46		Cause of learning disabilities: Fragile X syndrome	1009571000006104	1009571000006115
1009581000006117	EMISNQCA47		Cause of learning disabilities: Late effect of head injury	1009581000006101	1009581000006117
1009591000006119	EMISNQCA48		Cause of learning disabilities: Brain tumour	1009591000006103	1009591000006119
1009601000006110	EMISNQCA49		Cause of learning disabilities: Congenital hydrocephalus	1009601000006106	1009601000006110
1009611000006113	EMISNQCA50		Cause of learning disabilities: Microcephaly	1009611000006109	1009611000006113
1009621000006117	EMISNQCA51		Cause of learning disabilities: Phenylketonuria	1009621000006101	1009621000006117
1009631000006119	EMISNQCA52		Cause of learning disabilities: Prader-Willi syndrome	1009631000006103	1009631000006119
1009641000006112	EMISNQCA53		Cause of learning disabilities: Smith-Magenis syndrome	1009641000006108	1009641000006112
1009651000006114	EMISNQCA54		Cause of learning disabilities: Rett syndrome	1009651000006105	1009651000006114
1009661000006111	EMISNQCA55		Cause of learning disabilities: Congenital rubella	1009661000006107	1009661000006111
1009671000006116	EMISNQCA56		Cause of learning disablts: Unknown/awaiting investigation	1009671000006100	1009671000006116
1009681000006118	EMISNQCA57		Cause of learning disabilities: Unknown/despite investigation	1009681000006102	1009681000006118
1009691000006115	EMISNQCA58		Cause of learning disabilities: Other	1009691000006104	1009691000006115
1563991000006112	EMISNQLE3		Learning disability - specialty	1563991000006108	1563991000006112
1620441000006116	EMISNQLE5		Learning disability	1620441000006100	1620441000006116
1667711000000114	Eu86	Eu86.00	[X]Neurodevelopmental delay	751391000000106	1667711000000114
1667721000000115	Eu85	Eu85.00	[X]Global developmental delay	224958001	1667721000000115
1694831000006112	EMISNQPI5		Pitt-Hopkins syndrome	702344008	2995603016
1705901000006118	PKyz7-1	PKyz711	Angelman syndrome	76880004	1234038018
1786301000006110	PJ331-2	PJ33112	18q- syndrome	270889005	405347016
1786311000006113	PJ332-2	PJ33212	18p- syndrome	270890001	405348014
1786391000006115	PJ512-1	PJ51211	Duplication 10q syndrome	73035005	121311016
1786411000006115	PJ513-1	PJ51311	4p partial trisomy syndrome	49024004	81675017
1786421000006111	PJ514-1	PJ51411	9p partial trisomy syndrome	77527000	128694011
1786441000006116	PJ515-1	PJ51511	Duplication 15q syndrome	70324008	116814019
1786521000006110	PKyB-2	PKyB.12	Coloboma, heart malformation, choanal atresia, retardation of growth and development, genital abnormalities, and ear malformations (CHARGE) association	47535005	2620800011
1786571000006111	R034E	R034E00	Developmental delay	248290002	370667011
1798121000000114	P3423	P342300	Peters plus syndrome	449817000	2912534012
1798581000000119	PJ336	PJ33600	Chromosome 22q11 deletion syndrome	449818005	2912736012
1798621000000119	PJ338	PJ33800	Chromosome 4q deletion syndrome	37506004	2912992019
1800151000000118	PKyN	PKyN.00	Marden Walker syndrome	449824004	2912587012
1821361000006110	EMISNQIN133		Intellectual functioning disability	228156007	1821361000006110
1823961000006113	EMISNQLE13		Learning disability confirmed	1823961000006109	1823961000006113
1825141000006111	EMISNQAD62		Adrenoleucodystrophy	65389002	2154288010
1840501000006110	EMISNQSP29		Special educational needs - moderate learning difficulties	1840501000006106	1840501000006110
1840531000006119	EMISNQSP30		Special educ need-complex learning difficulties and disabilities	1840531000006103	1840531000006119

1872711000006116	EMISNQSP32		Special educational need type	1872711000006100	1872711000006116
1872721000006112	EMISNQSP33		Special education needs - specific learning disability	1872721000006108	1872721000006112
1872731000006110	EMISNQSP34		Special education needs - learning difficulty	1872731000006106	1872731000006110
1887331000006119	Eu818	Eu81800	Specific learning disability	889211000000104	2290291000000110
1939571000006115	14g00	14g0000	Early childhood developmental disability of unknown aetiology	954711000000106	2435131000000117
1947971000006116	Eu808	Eu80800	Developmental receptive language disorder	187921002	288890011
1947981000006118	Eu809	Eu80900	Acquired language comprehension impairment	716306006	3305472013
1947991000006115	Eu80A	Eu80A00	Developmental language comprehension impairment	716578009	3307207017
2107961000000111	PJ337	PJ33700	3p deletion syndrome	449819002	2168931000000118
2108161000000114	PJ331-3	PJ33113	18q deletion syndrome	270889005	2108161000000114
2108171000000119	PJ503-1	PJ50311	Trisomy 9 mosaic syndrome	74350000	123478012
2114791000000110	PJ33A	PJ33A00	Kleefstra syndrome	724207001	3432347016
2278791000000112	PJ54	PJ54.00	Ulnar mammary syndrome	700211007	2989585014
2278801000000111	PJ54-1	PJ54.11	Schinzel syndrome	700211007	2989657018
2288511000000116	13ZK0	13ZK000	Has statement of special educational needs	888421000000104	2288511000000116
2296351000000113	PJz31	PJz3100	MECP2 duplication syndrome	702816000	3006205015
2302801000000112	13Z4P	13Z4P00	Receiving learning support	894761000000108	2302801000000112
2388891000000118	14g0	14g0.00	Early childhood developmental disability	716710007	3307284014
2388901000000117	14g0-1	14g0.11	Early developmental impairment	716710007	3307285010
2417111000000110	1Bc0-1	1Bc0.11	Developmental dysarthria	230785002	2417111000000110
2435151000000112	13VC9	13VC900	Intellectual development disorder of unknown aetiology	954731000000103	2435151000000112
2508311000006110	^ESCTFR250831		FRAXA - Fragile X syndrome	613003	1232202017
2516621000006112	^ESCTDS251662		DSD - Developmental speech disorder	1145003	1220021016
2527721000006110	^ESCTMI252772		Microcephaly	1829003	4160017
2528311000006116	^ESCTDE252831		Developmental academic disorder	1855002	4203013
2528331000006110	^ESCTLE252833		Learning disorder	1855002	4208016
2528351000006115	^ESCTGE252835		General learning disability	1855002	478662017
2567771000006116	^ESCT11256777		11q partial monosomy syndrome	4325000	8287015
2589171000006110	^ESCTBA258917		Bardet-Biedl syndrome	5619004	10373017
2665621000006114	^ESCTLE266562		Lesch-Nyhan disease	10406007	272185011
2665631000006112	^ESCTHY266563		Hypoxanthine-guanine phosphoribosyltransferase deficiency	10406007	272186012
2670451000006119	^ESCTSP267045		Specific developmental disorder	10720004	18623010
2740491000000112	^ESCT1172259		Intellectual development disorder without significant impairment of behaviour	1094001000000106	2740491000000112
2740521000000110	^ESCT1172261		Intellectual development disorder with significant impairment of behaviour	1094011000000108	2740521000000110
2740551000000117	^ESCT1172263		Intellectual development disorder with minimal impairment of behaviour	1094021000000102	2740551000000117
2740581000000111	^ESCT1172265		Intellectual development disorder with impairment of behaviour	1094031000000100	2740581000000111
2771701000006114	^ESCT4P277170		4p partial monosomy syndrome	17122004	28958010
2782761000006112	^ESCTCR278276		Cross syndrome	17827007	30111010

2835541000006116	^ESCTCO283554	Complete trisomy 13 syndrome	21111006	35482019
2840101000006116	^ESCTDE284010	Developmental abnormality	21390004	1208681014
2923561000006111	^ESCTCA292356	Cat eye syndrome	26445008	44292015
3065731000006116	^ESCTTR306573	Trisomy X syndrome	35111009	58596018
3149211000006118	^ESCTDE314921	De Lange syndrome	40354009	63895010
3266991000006117	^ESCTME326699	Mental handicap	47437004	79090014
3267011000006111	^ESCTME326701	Mental subnormality	47437004	1230464013
3268621000006113	^ESCTCO326862	Coloboma, heart malformation, choanal atresia, retardation of growth and development, genital abnormalities, and ear malformations association	47535005	2914551016
3334491000006116	^ESCTCO333449	Complete trisomy 18 syndrome	51500006	85775018
3334501000006112	^ESCTED333450	Edwards syndrome	51500006	85776017
3462421000006114	^ESCTCU346242	Cutis laxa-corneal clouding-oligophrenia syndrome	59252009	98431015
3501751000006112	^ESCTAM350175	Amaurotic idiocy juvenile type	61663001	499087012
3514701000006113	^ESCTDE351470	Delayed articulatory and language development	62415009	103743015
3514711000006111	^ESCTDE351471	Developmental language delay	62415009	499275010
3562061000006117	^ESCTMU356206	Mucopolysaccharidosis, MPS-I-H	65327002	108537017
3562141000006117	^ESCTMU356214	Mucopolysaccharidosis type I-H	65327002	500080011
3563051000006112	^ESCTAD356305	Adrenoleukodystrophy	65389002	108634018
3563061000006114	^ESCTAD356306	Adrenomyeloneuropathy	65389002	108635017
3563111000006110	^ESCTAL356311	ALD - adrenoleukodystrophy	65389002	2957107010
3616531000006110	^ESCTRE361653	Rett's disorder	68618008	113976016
3616591000006114	^ESCTRE361659	Retts syndrome	68618008	2951769014
3629711000006110	^ESCTMU362971	Mucopolysaccharidosis type VI	69463008	501281010
3640931000006110	^ESCT5P364093	5p partial monosomy syndrome	70173007	116546014
3670581000006118	^ESCTCH367058	Childhood disintegrative disorder	71961003	119576018
3708621000006115	^ESCTCO370862	Complete trisomy 9 syndrome	74350000	123477019
3729521000006119	^ESCTMU372952	Mucopolysaccharidosis type I	75610003	503067012
3750051000006112	^ESCTAN375005	Angelman syndrome	76880004	127638013
3756821000006110	^ESCTBO375682	Borderline learning disability	77287004	503502012
3756851000006118	^ESCTBO375685	Borderline intellectual functioning	77287004	2951872010
3790911000006117	^ESCTLO379091	Lowe syndrome	79385002	131703019
3790971000006114	^ESCTCE379097	Cerebro-oculorenal dystrophy	79385002	1234350019
3811871000006110	^ESCTAI381187	Aicardi's syndrome	80651009	133795010
3828101000006111	^ESCTNE382810	Neurofibromatosis	81669005	135465015
3831011000006118	^ESCTAL383101	Alexander's disease	81854007	135788015
3831031000006112	^ESCTAL383103	Alexander disease	81854007	1234649011
4009971000006118	^ESCTNE400997	Neurofibromatosis 1	92824003	3009083017
4538731000006118	^ESCTLD453873	LD - Learning difficulties	161129001	251222017
4831001000006119	^ESCTTR483100	Trisomy 18 - meiotic nondisjunction	205623003	315354015
4831021000006112	^ESCTMO483102	Monosomy and deletion from autosome	205627002	315358017
4831061000006118	^ESCTWH483106	Whole chromosome monosomy - meiotic nondisjunction	205636003	315372014
4831091000006114	^ESCTWH483109	Whole chromosome trisomy syndrome	205646001	315388014

4831751000006117	^ESCTFO483175	Foetal alcohol syndrome	205788004	3010279011
4832001000006110	^ESCTNO483200	Noonan syndrome	205824006	2475676010
4926511000006114	^ESCTED492651	Educated at special needs school	224320009	337327011
4933261000006119	^ESCTSP493326	Special needs school	224863001	337985012
4935081000006116	^ESCTSL493508	Slow learner	224998004	338166012
4935091000006118	^ESCTLE493509	Learning delay	224998004	1222770016
4976271000006116	^ESCTSP497627	Special educational needs	228142005	342160013
4976281000006118	^ESCTSE497628	SEN - Special educational needs	228142005	342161012
4988761000006111	^ESCTSP498876	Special needs register	229056002	343386018
4998521000006113	^ESCTSP499852	Speech delay	229721007	344354016
4998611000006116	^ESCTDE499861	Developmental language impairment	229729009	344362012
4998671000006113	^ESCTEX499867	Expressive language delay	229734008	344368011
4998771000006115	^ESCTRE499877	Restricted language development	229741002	344376013
4998791000006119	^ESCTRE499879	Restricted receptive language development	229743004	344378014
5031301000006110	^ESCTLA503130	Laurence-Moon syndrome	232059000	347710015
5095461000006114	^ESCTPR509546	Prune belly syndrome with pulmonic stenosis, mental retardation and deafness	236529001	354548014
5116441000006113	^ESCTBE511644	Beta-D-mannosidosis	238047006	356823018
5129031000006113	^ESCTEC512903	Ectodermal dysplasia with hair-tooth-nail-sweating defect	239006001	358182016
5129141000006116	^ESCTEC512914	Ectodermal dysplasia with hair-tooth-nail defects	239015008	358194013
5129471000006118	^ESCTEC512947	Ectodermal dysplasia with nail defect	239046007	358233010
5245841000006110	^ESCTBE524584	Below average intellect	247575000	369677019
5340181000006114	^ESCTFR534018	FRAXA	254287005	378521015
5340191000006112	^ESCTFR534019	FRAXE	254288000	378522010
5517471000006110	^ESCTWH551747	Whole chromosome monosomy - mitotic nondisjunction mosaicism	270520003	405158019
5564871000006118	^ESCTDE556487	Delayed developmental milestone	274625009	2912590018
5591451000006112	^ESCTES559145	ESN - Educationally subnormal	276854003	413176017
5707211000006118	^ESCTSE570721	Severely educationally subnormal	285840007	425006015
5903381000006117	^ESCTFI590338	Finding relating to special educational needs	302141000	443698018
6459771000006113	^ESCTDE645977	Developmental articulation disorder	386701004	1480839016
6682941000006119	^ESCTOC668294	Oculo-cerebro-cutaneous syndrome (aplasia cutis, skin tags, eye & brain defects)	403554008	1782579015
6762781000006117	^ESCTLE676278	Learning disability - speciality	408468001	2154223015
6762801000006118	^ESCTLE676280	Learning disability	408468001	2163986010
7049741000006112	^ESCTCO704974	Cognitive developmental delay	425805004	2673542015
7265361000006111	^ESCTPR726536	Progressive encephalopathy with oedema, hypsarrhythmia and optic atrophy syndrome	442511009	2820530010
7281001000006112	^ESCTDE728100	Developmentally disabled	443656000	2838797017
7282231000006119	^ESCTNO728223	Nonverbal learning disorder	443735008	2842062017
7282241000006112	^ESCTNO728224	Nonverbal learning disability	443735008	2839181011
7295921000006119	^ESCTEX729592	Extra unidentified structurally abnormal chromosome	444655009	2870837017
7349161000006111	^ESCTFX734916	FXTAS - Fragile X associated tremor ataxia syndrome	448045004	1780771000000110

7375381000006117	^ESCTCH737538	Chromosome 3p deletion syndrome	449819002	2912647011
7495051000006114	^ESCTDE749505	Developmental regression	609225004	2958423017
7519841000006110	^ESCTOH751984	Ohdo syndrome, Maat-Kievit-Brunner type	699297004	2983700013
7519861000006114	^ESCTXL751986	X-linked Ohdo syndrome	699297004	2983727012
7519871000006119	^ESCTOH751987	Ohdo syndrome, Say-Barber-Biesecker-Young-Simpson variant	699298009	2983655015
7519891000006118	^ESCTSA751989	Say-Barber-Biesecker-Young-Simpson syndrome	699298009	2983642016
7525391000006116	^ESCTRE752539	Renpenning syndrome	699669001	2985573013
7534181000006118	^ESCTNE753418	Neurodevelopmental disorder	700364009	2989930017
7558081000006114	^ESCTAL755808	Allan-Herndon-Dudley syndrome	702327009	2995021019
7558801000006110	^ESCTCH755880	Chromosome 2q37 deletion syndrome	702357000	2995103015
7560021000006117	^ESCTPA756002	Partington syndrome	702412005	2995056015
7560111000006112	^ESCTSN756011	Snyder-Robinson syndrome	702416008	2995295014
7562541000006118	^ESCTDE756254	Developmental delay in receptive-expressive language	702528003	2995989017
7566981000006116	^ESCTME756698	Methyl-cytosine phosphate guanine binding protein-2 duplication syndrome	702816000	3006219015
7566991000006118	^ESCTME756699	Methyl-CpG (cytosine phosphate guanine) binding protein-2 duplication syndrome	702816000	3006199010
7574901000006110	^ESCTCA757490	CASK related intellectual disability	703389002	3008534018
7577041000006116	^ESCTNE757704	Neuronal ceroid lipofuscinosis 8	703526007	3009778015
7589061000006116	^ESCTIM758906	Impairment of child development	704304000	3012768013
7589071000006111	^ESCTIM758907	Impaired child development	704304000	3012776010
7589991000006111	^ESCTIM758999	Impaired infant development	704370008	3012989011
7730691000006117	^ESCTAL773069	Alpha thalassaemia X-linked intellectual disability syndrome	715342005	3302291017
7742511000006112	^ESCTEA774251	Early onset parkinsonism and intellectual disability syndrome	716107009	3304758012
7742581000006117	^ESCTKA774258	Kawashima Tsuji syndrome	716112005	3304777014
7743651000006117	^ESCTPE774365	Perniola Krajewska Carnevale syndrome	716191002	3305034010
7750931000006111	^ESCTFR775093	FRAXE intellectual disability syndrome	716709002	3307280017
7755141000006110	^ESCTL1775514	L1 syndrome	716996008	3308123013
7758091000006113	^ESCTXL775809	X-linked epilepsy with learning disability and behaviour disorder syndrome	717223008	3308721019
7769521000006118	^ESCTWO776952	Wolf Hirschhorn syndrome	718226002	3311589019
7774181000006117	^ESCTAC777418	Achalasia microcephaly syndrome	718573009	3312807014
7775921000006119	^ESCTOR777592	Oro-facial digital syndrome type 9	718680001	3313178014
7778251000006113	^ESCTFR777825	Fried syndrome	718848000	3314124019
7780351000006110	^ESCTXL778035	X-linked intellectual disability Schimke type	719010001	3314659018
7781071000006113	^ESCTSH778107	Shprintzen Goldberg craniosynostosis syndrome	719069008	3314858012
7781091000006114	^ESCTSH778109	Shprintzen-Goldberg syndrome	719069008	3314860014
7781471000006115	^ESCTBS778147	BSG syndrome	719097002	3314975017
7782161000006110	^ESCTXL778216	X-linked intellectual disability with cerebellar hypoplasia syndrome	719136005	3315132015
7782211000006118	^ESCTPE778221	Pettigrew syndrome	719139003	3315144017

7782501000006119	^ESCTXL778250	X-linked intellectual disability and hypotonia with facial dysmorphism and aggressive behaviour syndrome	719157002	3315196019
7782551000006115	^ESCTSY778255	Syndromic X-linked intellectual disability type 7	719160009	3315207010
7785901000006115	^ESCTMI778590	Microcephalus with brachydactyly and kyphoscoliosis syndrome	719378009	3316143016
7786681000006111	^ESCT15778668	15q11q13 microduplication syndrome	719427001	3316310010
7786891000006113	^ESCTDI778689	Disorder of sex development with intellectual disability syndrome	719450007	3316400017
7788481000006114	^ESCT16778848	16p13.11 microduplication syndrome	719578005	3316982015
7788521000006114	^ESCT17778852	17p13.3 microduplication syndrome	719582007	3316818012
7788761000006111	^ESCT19778876	19q13.11 microdeletion syndrome	719599008	3317093011
7789681000006118	^ESCT5Q778968	5q35 microduplication syndrome	719665003	3317303010
7791131000006110	^ESCTDO779113	DOORS syndrome	719800009	3321967011
7791151000006115	^ESCTDO779115	DOOR syndrome	719800009	3322824019
7791221000006119	^ESCTCH779122	Chromosome Xp11.3 microdeletion syndrome	719808002	3317983018
7791271000006118	^ESCTXL779127	X-linked intellectual disability Cabezas type	719811001	3317994019
7791631000006119	^ESCTWI779163	Wilson Turner syndrome	719834005	3318098016
7792761000006113	^ESCTTR779276	Trisomy Xq28 syndrome	719909009	3318425019
7792771000006118	^ESCTCH779277	Chromosome Xq28 trisomy syndrome	719909009	3318428017
7803961000006117	^ESCTAN780396	Aniridia and intellectual disability syndrome	720468000	3320943011
7804931000006111	^ESCTAU780493	Autosomal recessive limb girdle muscular dystrophy type 2K	720523006	3321129012
7806401000006115	^ESCTCE780640	Cerebro-facio-thoracic dysplasia	720635002	3321612013
7806471000006114	^ESCTCO780647	Coloboma, congenital heart disease, ichthyosiform dermatosis, intellectual disability ear anomaly syndrome	720639008	3321629011
7809711000006116	^ESCTFI780971	Filippi syndrome	720954000	3322630016
7809731000006110	^ESCTFI780973	Fine Lubinsky syndrome	720955004	3322635014
7809781000006111	^ESCTFO780978	Fountain syndrome	720957007	3322643016
7810331000006116	^ESCTAL781033	Alport syndrome, intellectual disability, midface hypoplasia, elliptocytosis syndrome	720982007	3322765013
7811761000006116	^ESCTDE781176	Deafness and intellectual disability Martin Probst type syndrome	721087008	3323440018
7811801000006113	^ESCTDE781180	Developmental delay, epilepsy, neonatal diabetes syndrome	721088003	3323445011
7813691000006118	^ESCTSE781369	Seizure, sensorineural deafness, ataxia, intellectual disability, electrolyte imbalance syndrome	721207002	3324614013
7826071000006112	^ESCTSC782607	Scholte syndrome	722002002	3330092011
7828621000006116	^ESCTSP782862	Spastic paraplegia, intellectual disability, palmoplantar hyperkeratosis syndrome	722209002	3331164011
7829441000006119	^ESCTAG782944	Agenesis of corpus callosum, intellectual disability, coloboma, micrognathia syndrome	722282008	3331425019
7831931000006118	^ESCTIN783193	Intellectual disability, developmental delay, contracture syndrome	722456001	3332213016
7832001000006117	^ESCTMA783200	Male hypergonadotropic hypogonadism, intellectual disability, skeletal anomaly syndrome	722459008	3332226017

7832211000006114	^ESCTSK783221	Skeletal dysplasia with intellectual disability syndrome	722478008	3332330016
7842691000006118	^ESCTIS784269	Isodicentric chromosome 15 syndrome	723332005	3424113016
7842701000006118	^ESCTDU784270	Duplication/inversion 15q11	723332005	3424114010
7842741000006116	^ESCTFA784274	Faciocardiorenal syndrome	723333000	3424121010
7850381000006114	^ESCTRE785038	Retinitis pigmentosa, intellectual disability, deafness, hypogenitalism syndrome	724001005	3481804013
7865911000006117	^ESCTTE786591	Temple Baraitser syndrome	725140007	3439393019
7870991000006110	^ESCTBU787099	Bullous dystrophy macular type	725589005	3444137014
7874821000006113	^ESCTIN787482	Intellectual disability Buenos Aires type	725906006	3446302016
7874881000006112	^ESCTNE787488	Neurofaciodigitorenal syndrome	725908007	3446330016
7877791000006114	^ESCTDU787779	Duplication of chromosome 3	726341009	3498271015
7881411000006110	^ESCTWE788141	Weaver Williams syndrome	726670008	3464489015
7881921000006114	^ESCTPR788192	Primrose syndrome	726709001	3451973010
7882141000006114	^ESCTXL788214	X-linked intellectual disability Nascimento type	726732002	3452277017
7952741000006112	^ESCTEP795274	Epilepsy, microcephaly, skeletal dysplasia syndrome	733031004	3498608019
7952761000006111	^ESCTEP795276	Epilepsy telangiectasia syndrome	733032006	3498611018
7953441000006114	^ESCTPS795344	Pseudoprogeria syndrome	733086003	3498717014
7957681000006110	^ESCTFA795768	Facial dysmorphism, macrocephaly, myopia, Dandy-Walker malformation syndrome	733417008	3499397016
7958391000006116	^ESCTMI795839	Microcephalus, glomerulonephritis, marfanoid habitus syndrome	733472005	3499508016
7958401000006119	^ESCT16795840	16p13.3 microduplication syndrome	733473000	3499511015
7958951000006114	^ESCT16795895	16p11.2p12.2 microduplication syndrome	733518000	3499592014
7959541000006113	^ESCTML795954	MLCRD (microcephaly with or without chorioretinopathy, lymphedema or intellectual disability) syndrome	733604003	3500041011
7963181000006117	^ESCTPO796318	Potocki Lupski syndrome	734016004	3481856017
8019831000006113	^ESCTMO801983	Moderate expressive language delay	62211000119103	2922262017
8019841000006115	^ESCTSE801984	Severe expressive language delay	62221000119105	2922281013
8019851000006118	^ESCTMI801985	Mild expressive language delay	62231000119108	2922254013
8026911000006110	^ESCTSE802691	Severe receptive language delay	89391000119105	2922263010
8026951000006111	^ESCTMI802695	Mild receptive language delay	89501000119108	2922240017
8056861000006118	^ESCTMI805686	Mixed receptive-expressive language delay	196901000000106	295681000000118
8195041000006117	^ESCTCA819504	Cause of learning disability	518831000000103	1158501000000112
8231171000006117	^ESCTNE823117	Neurodevelopmental delay	751391000000106	1653161000000110
8317111000006119	^ESCTSP831711	Speech and language developmental delay	898051000000104	2310281000000119
8337561000006119	^ESCTSI833756	Significant learning disability	931001000000105	2385981000000116
8337581000006112	^ESCTSI833758	Significant developmental disability	931001000000105	2714231000000111
8443531000006111	^ESCTDI844353	Difficulty learning basic skills	1070261000000101	2697211000000116
8443541000006118	^ESCTDI844354	Difficulty learning complex skills	1070271000000108	2697221000000110
11998101000006118	^ESCT1199810	Intellectual developmental disorder	110359009	3642975016
12000381000006119	^ESCT1200038	Charcot-Marie-Tooth disease, deafness, intellectual disability syndrome	763136000	3637979015
12000811000006118	^ESCT1200081	Grubben, De Cock, Borghgraef syndrome	763186006	3638112019

12002931000006115	^ESCT1200293	Cerebellar ataxia, intellectual disability, oculomotor apraxia, cerebellar cysts syndrome	763344007	3638624010
12007891000006110	^ESCT1200789	Wiedemann Steiner syndrome	763618001	3643042018
12009521000006116	^ESCT1200952	Intellectual disability, alacrima, achalasia syndrome	763741001	3643593016
12009551000006113	^ESCT1200955	Intellectual disability, spasticity, ectrodactyly syndrome	763743003	3643600019
12009571000006115	^ESCT1200957	Intellectual disability, brachydactyly, Pierre Robin syndrome	763744009	3643604011
12009911000006119	^ESCT1200991	Macrocephaly and developmental delay syndrome	763773007	3643793019
12010191000006115	^ESCT1201019	Malan overgrowth syndrome	763795006	3644015014
12010231000006113	^ESCT1201023	Agenesis of corpus callosum and abnormal genitalia syndrome	763797003	3644025016
12011241000006112	^ESCT1201124	Pachygyria, intellectual disability, epilepsy syndrome	763861000	3644781017
12016271000006113	^ESCT1201627	17q12 microduplication syndrome	764435003	3649828016
12016901000006114	^ESCT1201690	Mosaic trisomy 12	764463001	3649959018
12017691000006113	^ESCT1201769	Distal 22q11.2 microduplication syndrome	764524005	3650205012
12022541000006113	^ESCT1202254	Intellectual disability Birk-Barel type	764861005	3655432018
12023851000006118	^ESCT1202385	Cryptorchidism, arachnodactyly, intellectual disability syndrome	764950001	3655725014
12027071000006111	^ESCT1202707	Proximal 16p11.2 microduplication syndrome	765142003	3657168016
12027451000006111	^ESCT1202745	SCN8A-related epilepsy with encephalopathy	765170001	3657306014
12027461000006113	^ESCT1202746	SCN8A encephalopathy	765170001	3657302011
12047711000006118	^ESCT1204771	Nijmegen breakage syndrome-like disorder	766753005	3662497016
12049011000006112	^ESCT1204901	Diencephalic mesencephalic junction dysplasia	766871009	3662966013
12053971000006116	^ESCT1205397	22q11.2 deletion syndrome	767263007	3670124019
12077271000006118	^ESCT1207727	PPP2R5D-related intellectual disability	768677000	3686455015
12077811000006114	^ESCT1207781	15q13.3 microduplication syndrome	768713003	3686604015
12178711000006112	^ESCT1217871	Hyperphosphatasaemia with intellectual disability	33982008	3643091012
12197931000006114	^ESCT1219793	Borderline intellectual disability	77287004	3643525019
12204941000006113	^ESCT1220494	NF1 - Neurofibromatosis type 1	92824003	3671376015
12321891000006112	^ESCT1232189	Intellectual disability, congenital heart disease, blepharophimosis, blepharoptosis and hypoplastic teeth	412787009	3643126011
12337641000006113	^ESCT1233764	X-linked intellectual disability with marfanoid habitus	422437002	3643151012
12702211000006116	^ESCT1270221	Specific learning difficulty	889211000000104	2290321000000117
13491161000006116	^ESCT1349116	10q22.3q23.3 microdeletion syndrome	770401007	3700688012
13491351000006115	^ESCT1349135	Distal monosomy 19p13.3	770411000	3700744013
13491541000006114	^ESCT1349154	Early-onset epileptic encephalopathy and intellectual disability due to GRIN2A mutation	770431001	3700827018
13492091000006117	^ESCT1349209	Microcephalic primordial dwarfism Alazami type	770564004	3701291013
13492121000006118	^ESCT1349212	Monosomy 13q14 syndrome	770566002	3701297012
13492131000006115	^ESCT1349213	Deletion 13q14	770566002	3701299010
13492711000006113	^ESCT1349271	Ring chromosome 12 syndrome	770595006	3701491010
13493581000006116	^ESCT1349358	Tetrasomy 11q24.1	770663003	3701913015
13493791000006114	^ESCT1349379	Progressive encephalopathy with oedema, hypsarrhythmia, and optic atrophy-like syndrome	770678005	3702074016
13494421000006116	^ESCT1349442	3q27.3 microdeletion syndrome	770719004	3702236016

13494971000006112	^ESCT1349497	Intellectual disability, seizures, hypotonia, ophthalmologic, skeletal anomalies syndrome	770755007	3702470017
13497191000006119	^ESCT1349719	Autosomal recessive intellectual disability, motor dysfunction, multiple joint contracture syndrome	770901001	3703343018
13497201000006116	^ESCT1349720	Recessive intellectual disability, motor dysfunction, multiple joint contractures syndrome	770901001	3703344012
13497321000006119	^ESCT1349732	Kagami Ogata syndrome	770907002	3703367014
13497341000006114	^ESCT1349734	49,XXXYY syndrome	770908007	3703371012
13497811000006119	^ESCT1349781	Rhizomelic syndrome Urbach type	770948004	3703567013
13498421000006112	^ESCT1349842	Monosomy 9p	771072001	3704188015
13499591000006116	^ESCT1349959	Hepatic fibrosis, renal cyst, intellectual disability syndrome	771149000	3704579019
13501631000006113	^ESCT1350163	Polymicrogyria with optic nerve hypoplasia	771336003	3705808010
13501641000006115	^ESCT1350164	1q21.1 microduplication syndrome	771337007	3705813014
13503121000006119	^ESCT1350312	Developmental and speech delay due to SOX5 deficiency	771472009	3706361019
13503701000006111	^ESCT1350370	Autism spectrum disorder due to AUTS2 deficiency	771512003	3706567016
13510661000006110	^ESCT1351066	White Sutton syndrome	772127009	3717154011
13520601000006115	^ESCT1352060	Cyclin-dependent kinase-like 5 deficiency	773230003	3722626014
13520621000006113	^ESCT1352062	CDKL5 deficiency disorder	773230003	3722625013
13521771000006113	^ESCT1352177	Distal 7q11.23 microduplication syndrome	773325004	3723181013
13521831000006119	^ESCT1352183	CK syndrome	773329005	3723193016
13521841000006112	^ESCT1352184	X-linked intellectual disability, microcephaly, cortical malformation, thin habitus syndrome	773329005	3723194010
13522491000006113	^ESCT1352249	Autosomal recessive frontotemporal pachygyria	773394007	3723363018
13522641000006119	^ESCT1352264	Severe feeding difficulties, failure to thrive, microcephaly due to ASXL3 deficiency syndrome	773400009	3723393012
13522651000006117	^ESCT1352265	Bainbridge Roppers syndrome	773400009	3723394018
13522691000006111	^ESCT1352269	Intellectual disability with strabismus syndrome	773405004	3723417011
13523971000006111	^ESCT1352397	Autosomal recessive cerebellar ataxia, epilepsy, intellectual disability syndrome due to TUD deficiency	773498006	3723738016
13524851000006112	^ESCT1352485	Intellectual disability, craniofacial dysmorphism, cryptorchidism syndrome	773581009	3724281013
13525931000006114	^ESCT1352593	Distal Xq28 microduplication syndrome	773670004	3725602017
13529071000006110	^ESCT1352907	Peripheral dysostosis	773985008	3727471017
13530051000006113	^ESCT1353005	AHDC1-related intellectual disability, obstructive sleep apnoea, mild dysmorphism syndrome	774068004	3727864017
13530061000006110	^ESCT1353006	Xia Gibbs syndrome	774068004	3727860014
13621171000006117	^ESCT1362117	Distal trisomy 18q	782676009	3755116014
13622051000006117	^ESCT1362205	Intellectual disability, facial dysmorphism syndrome due to SETD5 haploinsufficiency	782736007	3755540013
13622301000006119	^ESCT1362230	Intellectual disability, coarse face, macrocephaly, cerebellar hypotrophy syndrome	782753000	3755618014
13622321000006112	^ESCT1362232	Autosomal recessive spinocerebellar ataxia type 20	782753000	3755620012

13622561000006113	^ESCT1362256	Congenital muscular dystrophy with intellectual disability and severe epilepsy	782772000	3755710013
13622571000006118	^ESCT1362257	Congenital disorder of glycosylation type 1u	782772000	3755714016
13622581000006115	^ESCT1362258	Carbohydrate deficient glycoprotein syndrome type 1u	782772000	3755716019
13624181000006116	^ESCT1362418	Infantile spasms, psychomotor retardation, progressive brain atrophy, basal ganglia disease syndrome	782886007	3756554010
13625421000006113	^ESCT1362542	Severe microbrachycephaly, intellectual disability, athetoid cerebral palsy syndrome	783005002	3757107011
13627911000006119	^ESCT1362791	Congenital muscular dystrophy with intellectual disability	783174004	3757954011
13630921000006112	^ESCT1363092	Has special educational needs	783572008	3759384019
13631361000006113	^ESCT1363136	DYRK1A-related intellectual disability syndrome due to 21q22.13q22.2 microdeletion	783619003	3759527011
13632231000006117	^ESCT1363223	White matter hypoplasia, corpus callosum agenesis, intellectual disability syndrome	783703004	3759781012
13662101000006111	^ESCT1366210	Developmental delay, facial dysmorphism syndrome due to MED13L deficiency	787093004	3773933016
13697811000006116	^ESCT1369781	Significant learning disability	1239331000000100	2800231000000118
13784441000006119	^ESCT1378444	Learning disability	110359009	2800271000000116
13904831000006116	^ESCT1390483	Alopecia, epilepsy, intellectual disability syndrome Moynahan type	788417006	3780616016
13939301000006114	^ESCT1393930	MASA syndrome	838441009	3896942012
13963361000006115	^ESCT1396336	Pervasive developmental disorder with disorder of intellectual development without loss of previously acquired skills	870262000	3968748015
13963421000006115	^ESCT1396342	Pervasive developmental disorder with disorder of intellectual development with loss of previously acquired skills	870265003	3968757014
13963431000006117	^ESCT1396343	Autism spectrum disorder with disorder of intellectual development and with mild or no impairment of functional language with loss of previously acquired skills	870265003	3968756017
13963441000006110	^ESCT1396344	Pervasive developmental disorder with disorder of intellectual development and marked impairment of functional language with loss of previously acquired skills	870266002	3968759012
13963451000006112	^ESCT1396345	Autism spectrum disorder with disorder of intellectual development and with impaired functional language with loss of previously acquired skills	870266002	3968761015
13963461000006114	^ESCT1396346	Pervasive developmental disorder with disorder of intellectual development and marked impairment of functional language without loss of previously acquired skills	870267006	3968763017
13963471000006119	^ESCT1396347	Autism spectrum disorder with disorder of intellectual development and impaired functional language without loss of previously acquired skills	870267006	3968762010
13963481000006116	^ESCT1396348	Pervasive developmental disorder with disorder of intellectual development and complete impairment of functional language without loss of previously acquired skills	870268001	3968767016

13963491000006118	^ESCT1396349	Autism spectrum disorder with disorder of intellectual development and complete impairment of functional language without loss of previously acquired skills	870268001	3968765012
13963521000006116	^ESCT1396352	Pervasive developmental disorder with disorder of intellectual development and complete impairment of functional language with loss of previously acquired skills	870270005	3968771018
13963531000006118	^ESCT1396353	Autism spectrum disorder with disorder of intellectual development and complete impairment of functional language with loss of previously acquired skills	870270005	3968773015
13964061000006119	^ESCT1396406	Pervasive developmental disorder with cognitive developmental delay and marked impairment of functional language	870305003	3968864019
13964071000006114	^ESCT1396407	Autism spectrum disorder with disorder of intellectual development and impaired functional language	870305003	3968863013
13964131000006112	^ESCT1396413	Autism spectrum disorder with disorder of intellectual development and complete impairment of functional language	870308001	3968871012
14072761000006111	^ESCT1407276	Hennekam syndrome	234146006	3789477016
14132561000006113	^ESCT1413256	Bilateral megalencephaly	879919001	3993786019
14134231000006111	^ESCT1413423	12q15 deletion syndrome	880081006	3994444018
14135491000006110	^ESCT1413549	3p25.3 deletion syndrome	890123006	4011205015
14135571000006113	^ESCT1413557	9q34 deletion syndrome	890130000	4009227018
14137521000006116	^ESCT1413752	Bilateral frontal polymicrogyria	890285006	4011911011
14137531000006118	^ESCT1413753	Bilateral frontoparietal polymicrogyria	890286007	4011914015
14152591000006118	^ESCT1415259	Molybdenum cofactor deficiency complementation group B	1003368009	4168093015
14152831000006119	^ESCT1415283	Mosaic 1q duplication	1003389000	4168139015
14153041000006114	^ESCT1415304	Maternal 15q11q13 deletion	1003409002	4168186016
14163361000006116	^ESCT1416336	X-linked complicated corpus callosum dysgenesis	1010630006	4213373017
14432361000006117	^ESCT1443236	Psychomotor retardation	1144814003	4543404012
14441711000006111	^ESCT1444171	Microcephaly	1148757008	4551753012
14441721000006115	^ESCT1444172	Microcephalus	1148757008	4551752019
14452321000006112	^ESCT1445232	Partial deletion of short arm of chromosome 5	1153583005	4565146017
14507171000006113	^ESCT1450717	Oculocerebrocutaneous syndrome	403554008	4579831016
14512561000006111	^ESCT1451256	Trisomy Xq28	719909009	4570723012
14549611000006112	^ESCT1454961	Brain malformations, musculoskeletal abnormalities, facial dysmorphism, intellectual disability syndrome	1169355000	4607740010
14552291000006113	^ESCT1455229	Early-onset epilepsy, intellectual disability, brain anomalies syndrome	1172627007	4634848019
14552331000006118	^ESCT1455233	TBCK-related intellectual disability syndrome	1172628002	4634855017
14552351000006113	^ESCT1455235	Severe growth deficiency, strabismus, extensive dermal melanocytosis, intellectual disability syndrome	1172629005	4634867015
14553091000006112	^ESCT1455309	Global developmental delay, visual anomalies, progressive cerebellar atrophy, truncal hypotonia syndrome	1172696009	4635425014
14564831000006111	^ESCT1456483	DYRK1A-related intellectual disability syndrome	1179301003	4650644016

14566451000006113	^ESCT1456645	CHD3-related developmental delay, speech delay, intellectual disability, abnormalities of vision, facial dysmorphism syndrome	1179408008	4651034011
14576241000006115	^ESCT1457624	STAG1-related intellectual disability, facial dysmorphism, gastrooesophageal reflux syndrome	1187041000	4669042011
14577761000006112	^ESCT1457776	Intellectual disability, epilepsy, extrapyramidal syndrome	1187210007	4672728014
14578491000006112	^ESCT1457849	Macrocephaly, intellectual disability, neurodevelopmental disorder, small thorax syndrome	1187304005	4673265016
14608951000006111	^ESCT1460895	Tetrasomy 12p syndrome	9527009	4589824013
14753781000006110	^ESCT1475378	PHIP-related behavioural problems, intellectual disability, obesity, dysmorphic features syndrome	1208987006	5013861019
14770551000006118	^ESCT1477055	Neurodevelopmental disorder, craniofacial dysmorphism, cardiac defect, skeletal anomalies syndrome	1222710008	5048626018
14790031000006114	^ESCT1479003	CNTNAP2-related developmental and epileptic encephalopathy	1230376005	5069594018
15021671000006111	^ESCT1502167	Autosomal dominant intellectual disability, craniofacial anomalies, cardiac defects syndrome	1255319004	5146300011
15047751000006115	^ESCT1504775	GRIN2B-related developmental delay, intellectual disability, autism spectrum disorder	1260195002	5159667013
15080241000006115	^ESCT1508024	Prune belly syndrome with pulmonic stenosis, intellectual disability and deafness	236529001	5155337016
15242911000006112	^ESCT1524291	Developmental and epileptic encephalopathy	1275631007	5224368014

Supplementary table 2. A list of codes to identify symptoms testing of potentially indicative of bowel cancer.

OriginalReadCode	CleansedReadCode	Term	SnomedCTConceptId	SnomedCTDescriptionId
J5730-1	J573011	Rectal bleeding	12063002	20792019
19C	19C..00	Constipation	14760008	25076018
1969	1969.00	Abdominal pain	21522001	36112013
19F2	19F2.00	Diarrhoea	62315008	103578017
197A	197A.00	Generalised abdominal pain	102614006	252305018
1963	1963.00	Non-colicky abdominal pain	162038003	252571013
1971	1971.00	Central abdominal pain	162046002	252584016
1977	1977.00	Right iliac fossa pain	162051008	252589014
1978	1978.00	Left iliac fossa pain	162052001	252594014
25C6	25C6.00	On examination - abdominal pain - umbilical	163218001	2667728011
25C8	25C8.00	On examination - abdominal pain - right iliac	163220003	2667730013
25J	25J..00	O/E - abdominal mass palpated	163278007	254422016
25J2	25J2.00	On examination - abdominal mass < 1 quadrant	163280001	2667767017
25J3	25J3.00	On examination - abdominal mass fills 1 quadrant	163281002	2667768010
25J4	25J4.00	On examination - abdominal mass fills half abdomen	163282009	2667769019
25J5	25J5.00	On examination - abdominal mass fills abdomen	163283004	2667770018
25J7	25J7.00	Right iliac fossa mass	163285006	254429013
25JZ	25JZ.00	O/E - abd. mass palpated NOS	163278007	254422016
25K	25K..00	O/E-abdominal mass consistency	163290009	254434012
25K1	25K1.00	O/E - abdominal mass - soft	163291008	254435013
25K2	25K2.00	O/E - abdominal mass - hard	163292001	254436014
25K3	25K3.00	O/E - abdominal mass-very hard	163293006	254437017
25KZ	25KZ.00	O/E - abd.mass consistency NOS	163290009	254434012
25L	25L..00	O/E - abdominal mass shape	163296003	254440017
25L1	25L1.00	On examination - abdominal mass - regular shape	163297007	2667779017
25L2	25L2.00	On examination - abdominal mass - irregular shape	163298002	2667780019
25LZ	25LZ.00	O/E - abd. mass shape NOS	163296003	254440017
25M2	25M2.00	On examination - abdominal mass still with respiration	163303000	2666813012
25MZ	25MZ.00	O/E - abd.mass + respn. NOS	163302005	254447019
25N	25N..00	On examination - abdominal mass -border defined	163305007	2666815017
25N1	25N1.00	On examination - abdominal mass - upper border defined	163306008	2666816016
25N2	25N2.00	On examination - abdominal mass - lower border defined	163307004	2667645011
25NZ	25NZ.00	O/E -abd.mass -border def. NOS	163305007	254450016
25Q3	25Q3.00	O/E - PR - rectal mass	163326007	254477013

25R2	25R2.00	On examination - tympany over abdominal mass	163335000	2668526011
25R3	25R3.00	On examination - dullness over abdominal mass	163336004	2668527019
264	264..00	O/E - pelvic mass palpated	163367002	254524018
2641	2641.00	O/E - pelvic mass palpable-LIF	163368007	254525017
2642	2642.00	O/E - pelvic mass palpable-RIF	163369004	254526016
2643	2643.00	O/E - central pelvic mass	163370003	254527013
264Z	264Z.00	O/E - pelvic mass palpable NOS	163367002	254524018
D00yz	D00yz00	Iron deficiency anemia	87522002	507616014
D00z	D00z.00	Unspecified iron deficiency anaemia	87522002	507616014
D00zz	D00zz00	Iron deficiency anaemia NOS	87522002	507616014
Dyu00	Dyu0000	[X]Other iron deficiency anaemias	87522002	507616014
J5200	J520000	Acute constipation	197119006	303162012
J520y	J520y00	Other specified constipation	14760008	25076018
19CZ	19CZ.00	Constipation NOS	14760008	25076018
R030	R030.00	[D]Anorexia	496871000000107	1105981000000113
R030z	R030z00	[D]Anorexia NOS	79890006	132549012
R0771	R077100	Loose stools	398032003	1786047017
R078	R078.00	[D]Change in bowel habit	88111009	507901013
R090	R090.00	[D]Abdominal pain	21522001	36112013
R0906	R090600	Umbilical pain	88522004	146777018
R0909	R090900	Pain in right iliac fossa	162051008	252591018
R090A	R090A00	Pain in left iliac fossa	162052001	252592013
R090y	R090y00	[D]Other specified abdominal pain	21522001	36112013
R090z	R090z00	[D]Abdominal pain NOS	21522001	36112013
R093	R093.00	Intra-abdominal and pelvic swelling, mass and lump	274719002	410563016
R0932	R093200	Abdominal lump	271860004	406852014
R0933	R093300	Pelvic swelling	274754009	410615013
R0934	R093400	Pelvic mass	74285003	123367017
R0935	R093500	[D]Pelvic lump	74285003	123367017
R0937	R093700	[D]Umbilical mass	274758007	410620013
R0938	R093800	Umbilical mass	274758007	410620013
R0939	R093900	Groin swelling	274743004	410596019
R093A	R093A00	Groin mass	281398003	1208718019
R093B	R093B00	Groin lump	281398003	419394010
R093z	R093z00	[D]Swelling, mass or lump within abdomen or pelvis NOS	274719002	410563016
Ryu11	Ryu1100	[X]Other and unspecified abdominal pain	21522001	36112013
J5204	J520400	Chronic constipation	236069009	353853017
J43z-1	J43z.11	Chronic diarrhoea	236071009	353856013
197-2	197..12	Iliac fossa pain	247354009	369362015

196-1	196..11	Abdominal pain type	247358007	369368016
1612-1	1612.11	Anorexia symptom	249468005	372208017
1625	1625.00	Abnormal weight loss	267024001	397867018
19F	19F..00	Diarrhoea symptoms	267060006	397927015
19FZ	19FZ.00	Diarrhoea symptom	267060006	2575888019
D00y	D00y.00	Iron deficiency anemia	87522002	507616014
D21z	D21z.00		271737000	406638014
1612	1612.00	Appetite loss - anorexia	249468005	372208017
R0931	R093100	Abdominal mass	271860004	406851019
R090L	R090L00	Left lower quadrant pain	301716002	443111010
R090M	R090M00	Right lower quadrant pain	301754002	443197010
R090N	R090N00	Nonspecific abdominal pain	304542004	446863014
25J8	25J8.00	O/E left lower abdominal mass	312355005	456056015
J5730-2	J573012	PRB - Rectal bleeding	12063002	464499018
J5730	J573000	Rectal haemorrhage	12063002	464500010
J5201	J520100	Chronic constipation with overflow	31499008	484894013
D00y1	D00y100	Microcytic hypochromic anaemia	44666001	493975011
19EA	19EA.00	Change in bowel habit	88111009	507901013
19EA-1	19EA.11	Altered bowel habit	88111009	507902018
197A-1	197A.11	General abdominal pain-symptom	102614006	1218836019
22A8	22A8.00	Weight loss from baseline weight	401003006	1780210016
19F-2	19F..12	Loose stool	398032003	1777604018
19E6-2	19E6.12	Haematochezia	405729008	2153991014
19C2	19C2.00	Constipated	14760008	2162207016
196B	196B.00	Painful rectal bleeding	414991007	2534219010
196C	196C.00	Painless rectal bleeding	414992000	2534250012
D21	D21..00	Other and unspecified anaemias	271737000	406638014
J4z-1	J4z..11	Presumed non-infectious diarrhoea	69980003	1216880010
J4-3	J4...13	Non-infective diarrhoea	69980003	501455014
1D1A	1D1A.00	Complaining of weight loss	198511000000103	299281000000119
1625-1	1625.11	Abnormal weight loss - symptom	267024001	397867018
J5731	J573100	Bleeding from anus	6072007	498827015
J573-1	J573.11	Bleeding per rectum	12063002	464494011
J520z	J520z00	Constipation NOS	14760008	25076018
19C-1	19C..11	Constipation symptom	14760008	25076018
19F-1	19F..11	Diarrhoea	62315008	103578017
J4zz-1	J4zz.11	Diarrhoea - presumed non-infectious	25374005	42550011
D00-2	D00..12	Microcytic - hypochromic anaemia	44666001	493975011
D00	D00..00	Iron deficiency anaemia	87522002	507616014

D00-1	D00..11	Hypochromic - microcytic anaemia	44666001	493975011
J573-99	J573.99	Anal/rectal haemorrhage	266464001	886471000006110
1623-99	1623.99	Weight Loss	161832001	903061000006114
HNG0080		[RFC] Constipation	906171000006108	906171000006112
HNG0131		[RFC] Iron deficiency anaemia	906571000006103	906571000006119
HNG0634		[RFC] Constipation	909031000006103	909031000006119
HNGZ003		[RFC] Loose stools	909311000006106	909311000006110
EMISNQCO9		Colicky abdominal pain present	958291000006108	958291000006112
EMISCAN3		Anorexia	960281000006104	960281000006115
EMISCUN9		Unexplained/progressive weight loss	960561000006106	960561000006110
EMISCCO20		Constipation	982721000006105	982721000006114
EMISCDI58		Diarrhoea/loose stools	982731000006108	982731000006112
EMISCCH2		Change in bowel habit	982741000006103	982741000006119
EMISNQBL1		Bloody diarrhoea	95545007	201773015
1627	1627.00	Unintentional weight loss	448765001	2899697019
EMISNQCH56		Chronic constipation	1726521000006104	1726521000006115
EMISNQRI7		Right upper quadrant mass	1805241000006109	1805241000006113
EMISNQLE10		Left upper quadrant mass	1805251000006106	1805251000006110
EMISNQLE11		Left iliac fossa mass	307135001	450317013
EMISNQCE19		Central abdominal mass	404200001	2156097019
^ESCTSP265912		Spasmodic abdominal pain	9991008	195250015
^ESCTCO265913		Colicky abdominal pain	9991008	1236016018
^ESCTPR269200		PR - Bleeding per rectum	12063002	464495012
^ESCTRB269203		RB - Rectal bleeding	12063002	464498014
^ESCTRE269205		Rectal hemorrhage	12063002	464501014
^ESCTCN273389		CN - Constipation	14760008	2162208014
^ESCTCH277850		Chronic diarrhoea of unknown origin	17551007	195832016
^ESCTAP284225		AP - Abdominal pain	21522001	481053019
^ESCTMI322021		Microcytic hypochromic anemia	44666001	74505017
^ESCTHY322022		Hypochromic microcytic anemia	44666001	74506016
^ESCTHY322023		Hypochromic microcytic anaemia	44666001	493976012
^ESCTDI351272		Diarrhea	62315008	103576018
^ESCTDD351273		D - Diarrhoea	62315008	499248015
^ESCTDD351274		D - Diarrhea	62315008	499249011
^ESCTNO363778		Non-infective diarrhea	69980003	501454013
^ESCTPR363780		Presumed non-infectious diarrhea	69980003	1218367013
^ESCTPE370768		Pelvic lump	74285003	123372014
^ESCTAN378538		Anaemia due to unknown mechanism	79035003	504005012
^ESCTAN379925		Anorexia	79890006	132550012

^ESCTID392269	IDA - Iron deficiency anemia	87522002	507617017
^ESCTID392272	IDA - Iron deficiency anaemia	87522002	507620013
^ESCTAL393194	Altered bowel habits	88111009	146080012
^ESCTWE395253	Weight loss	89362005	148168013
^ESCTHA405831	Haemorrhagic diarrhoea	95545007	512178019
^ESCTHE405832	Hemorrhagic diarrhea	95545007	158254017
^ESCTBL405833	Bloody diarrhea	95545007	158255016
^ESCTLO407773	Localised abdominal pain	102613000	252289013
^ESCTLO407774	Localized abdominal pain	102613000	165941018
^ESCTGE407776	Generalized abdominal pain	102614006	165942013
^ESCTAC427038	Acute abdominal pain	116290004	179236013
^ESCTDI440738	Diarrhoeal disorder	128333008	1205543012
^ESCTDI440740	Diarrheal disorder	128333008	474373013
^ESCTDI440741	Diarrhoeal disease	128333008	474374019
^ESCTPR454471	Progressive weight loss	161832001	2646366011
^ESCTRI454620	RIF - Right iliac fossa pain	162051008	252590017
^ESCTLI454624	LIF - Left iliac fossa pain	162052001	252593015
^ESCTPA454626	Pain of hypogastrium	162053006	252596011
^ESCTOE456122	O/E - abdominal mass - irregular shape	163298002	2667781015
^ESCTON456162	On examination - per rectum - rectal mass	163326007	2668513012
^ESCTPR508934	Protracted diarrhoea	236077008	353872017
^ESCTRE525850	Rectal mass	248523006	370977010
^ESCTCO527277	Constipation alternates with diarrhoea	249517009	372278016
^ESCTCO527278	Constipation alternates with diarrhea	249517009	372279012
^ESCTCO556147	C/O right iliac fossa pain	274277005	410058011
^ESCTCO556148	Complaining of right iliac fossa pain	274277005	2669676010
^ESCTCO556149	C/O left iliac fossa pain	274278000	410059015
^ESCTCO556150	Complaining of left iliac fossa pain	274278000	2669606014
^ESCTON556165	On examination - abdominal pain	274287009	2669894018
^ESCTUM556600	Umbilical lump	274758007	2900324016
^ESCTLE570065	Left sided abdominal pain	285387005	424372018
^ESCTRI570066	Right sided abdominal pain	285388000	424373011
^ESCTVI587951	Visible abdominal mass	300404004	441469019
^ESCTIL596479	Iliac fossa abdominal mass	307134002	450316016
^ESCTEX598663	Excessive weight loss	309257005	452625013
^ESCTON601534	On examination - left lower abdominal mass	312355005	2670657017
^ESCTAB604382	Abdominal pain - cause unknown	314212008	458531010
^ESCTUN604383	Unexplained abdominal pain	314212008	458532015
^ESCTAN604793	Anterior abdominal wall mass	314603004	458985019

^ESCTLS659646	LS - Loose stools	398032003	1786048010
^ESCTSE678095	Severe diarrhoea	409587002	2469135010
^ESCTSE678096	Severe diarrhea	409587002	2469609013
^ESCTUN699737	Unexplained weight loss	422868009	2645676012
^ESCTRE706719	Recent weight loss	426977000	2676113010
^ESCTPE727850	Periumbilical pain	443503005	2839848012
^ESCTPE727851	Periumbilical abdominal pain	443503005	2873027010
^ESCTIN735984	Involuntary weight loss	448765001	3425621016
^ESCTIN735997	Intra-abdominal mass	448772000	2899436018
^ESCTCO762060	Continuous abdominal pain of left lower quadrant	707597009	3030682018
^ESCTMA785513	Mass of abdominal wall	724388006	3434991014
^ESCTAB785514	Abdominal wall mass	724388006	3434992019
^ESCTLE801625	Left lower quadrant abdominal swelling, mass, or lump	39261000119104	3015460014
^ESCT1271767	[D]Anorexia	496871000000107	1105991000000110
^ESCT1276169	Iron deficiency anemia	87522002	145104011
^ESCT1392363	Weight loss	816160009	3850507018
^ESCT1450753	Painful rectal haemorrhage	414991007	4543166018
^ESCT1516692	Anal mass	1812681000000102	3390361000000112

Supplementary table 4. A list of codes to identify faecal immunochemical test and faecal occult blood tests.

OriginalReadCode	CleansedReadCode	Term	SnomedCTConceptId	SnomedCTDescriptionId
479	479..00	Faecal occult blood test	1015401000000102	2574791000000110
4792	4792.00	Faecal occult blood: negative	167667006	260506013
4794	4794.00	Occult blood in stools	59614000	99030019
479Z	479Z.00	Screening for occult blood in faeces	104435004	277449011
4793	4793.00	Faecal occult blood: trace	389076003	1476223019
6887	6887.00	OB - Occult blood screening	252156002	375535019
68W2	68W2.00	Bowel cancer screening programme	286901000000107	495731000000114
4795	4795.00	Serial faecal occult blood normal	320531000000106	586041000000119
4796	4796.00	Serial faecal occult blood abnormal	320581000000105	586141000000118
479-1	479..11	Faeces occult blood test	1015401000000102	2574791000000110
6866	6866.00	Bowel cancer screening programme: faecal occult blood result	368481000000103	714661000000115
EMISNQBO16		Bowel cancer screening - positive FOBs	1626191000006103	1626191000006119
EMISNQBO17		Bowel cancer screening - negative FOBs	1626201000006100	1626201000006116

6869	6869.00	Bowel cancer screening programme faecal occult blood test result unclear	375181000000107	737871000000112
686A	686A.00	Bowel cancer screening programme faecal occult blood test normal	375211000000108	737931000000118
686B	686B.00	Bowel cancer screening programme faecal occult blood test abnormal	375241000000109	737991000000117
47K		Quantitative faecal immunochemical test	1049361000000101	2643291000000112
^ESCTOC346835		Occult blood in stool	59614000	2920745016
^ESCTFA410673		Faecal occult blood screen	104435004	1215844018
^ESCTFA410674		Faecal occult blood screening	104435004	1215845017
^ESCTFO410675		FOB - Faecal occult blood screening	104435004	1215846016
^ESCTFO410677		FOB - Fecal occult blood screening	104435004	1217345010
^ESCTFE410678		Fecal occult blood screen	104435004	1217346011
^ESCTFE460598		Fecal occult blood: negative	167667006	260505012
^ESCTOC531127		Occult blood screening	252156002	375534015
^ESCTBO812602		Bowel cancer screening programme finding	384171000000104	758021000000118
^ESCTFA839624		Faeces occult blood test	1015401000000102	2565621000000114
^ESCTQF842594		QFIT - Quantitative faecal immunochemical test	1049361000000101	2643321000000119
^ESCT1211944		Bowel cancer screening programme liquid faecal immunochemical test normal	1101361000000103	2757041000000113
^ESCT1211945		Bowel cancer screening programme liquid faecal immunochemical test abnormal	1101371000000105	2757061000000114
^ESCT1219009		Faecal occult blood positive	59614000	3638412012
^ESCT1219010		Fecal occult blood positive	59614000	3638413019
^ESCT1276207		Screening for occult blood in faeces	104435004	12762071000006115
^ESCT1530727		Occult blood detected in faeces	59614000	5171884016
^ESCT1531042		Occult blood not detected in faeces	167667006	5171878019
^ESCT1531739		Trace occult blood detected in faeces	389076003	5171887011
